# Supplementary material for: Aortic stenosis post-COVID-19: a mathematical model on waiting lists and mortality
Source: BMJ Open. 2022 Jun 16;12(6):e059309. doi: 10.1136/bmjopen-2021-059309 (PMC9207579; doi:10.1136/bmjopen-2021-059309)
Supplement: Supplementary data [file bmjopen-2021-059309supp001.pdf]

SUPPLEMENTS

Supplement 1: Mathematical Derivation of the Differential Equation and its Solution

From figure 1, we can write the following equation:

$$\frac{dW}{dt} = f - r_T - r_S - \mu W.$$

We can then re-write and integrate this equation

$$\int_0^{t_c} 1dt = \int_{W_0}^{0} \frac{1}{f - r_T - r_S - \mu W} dW$$

$$t_c = \left[-\frac{1}{\mu} ln\,(f - r_T - r_s - \mu W)\right]_{W_0}^{0} = \left[\frac{1}{\mu} ln\,(f - r_T - r_s - \mu W)\right]_{0}^{W_0}.$$

We can now define T_e, the extra capacity, as $T_e = r_T + r_S - f$. This is because we claim that under normal conditions, $f = r_T^0 + r_S^0$, such that the waiting list never grows above zero, and that the additional patients are already on the waiting list. The equation for T_e follows the observation that the current rates of TAVI and SAVR treatment are the normal rates plus the additional capacity.

This substitution allows us to write

$$t_c = \frac{1}{\mu}(ln\,(-T_e - \mu W_0)\; - ln\,(-T_e)\,) = ln\left(1 + \frac{\mu W_0}{T_e}\right)\mu^{-1}.$$

This is the solution we use for calculating the time when the waiting list becomes zero.

We now rely on the assumption that T_e is constant to write

$$m(t_c) = W_0 - T_e t_c.$$

That is, by the time the waiting list is zero, everyone who has not been treated is unfortunately dead.

The assumption of a front-loaded waiting list (i.e., that all additional patients are identified and waiting) is not a strict requirement for this model to be valid. If it is the case that the additional patients are still being identified when the extra capacity is created, then as long as

they are identified at a faster rate than they are treated, the predictions in this model hold. It is only in cases where the identification rate is less than the treatment rate that this assumption becomes invalid. In such cases, T_e can be said to be equal to the identification rate instead. This is true because mortality is not tied to being on the waiting list but from the onset of symptoms. In this way, the waiting list in our model can be thought of as the list of all people who need treatment, even if the NHS is unaware of them.

This model can be extended to predict mortality and time to clear a waiting list for non-constant T_e, but we do not expand on that here.

Supplement 2: Data

We calculate the increase in capacity due to conversions and operational changes as follows. Assume that we increase operations by 20% due to operational changes and convert 10% of all SAVR to TAVI. Also assume that for every three SAVR patients five TAVI patients can be processed. If we convert 10% of SAVR cases to TAVI (783 SAVR patients), we can treat an additional 522 patients from the waiting list. From the 20% increase, we get extra 1039 TAVI and 1566 SAVR operations per year. If we apply 10% conversion to this extra capacity, 156 SAVR operations can be converted into 260 TAVI operations. In total, the operational changes and conversion create an extra capacity of 3232 operations with which to service the waiting list each year: 1822 (1,039+522+261) TAVI and 1410 (1,566-156) SAVR operations.

N.B. We make no assumptions about who the extra TAVI procedures treat, for example, if in the above example, the additional 626 TAVI procedures we gain from conversion (522 from converting the normal capacity and 104 from converting the additional capacity) treated only SAVR patients, the conversion rate would actually be $\frac{626+783+156}{626+1566+7830} = 15.6\%$. Normally, we would expect that the application of this extra TAVI would be in the same proportion as the ratio of SAVR to TAVI, which would give a real-world conversion rate of 13.5%.

Supplement 3: App

The app can be accessed at https://github.com/Christian-P-Stickels/AS_Waitinglist_data

Supplement 4: Additional Results

Supplementary figure S1: Heat map of a three-to-four SAVR-to-TAVI conversion

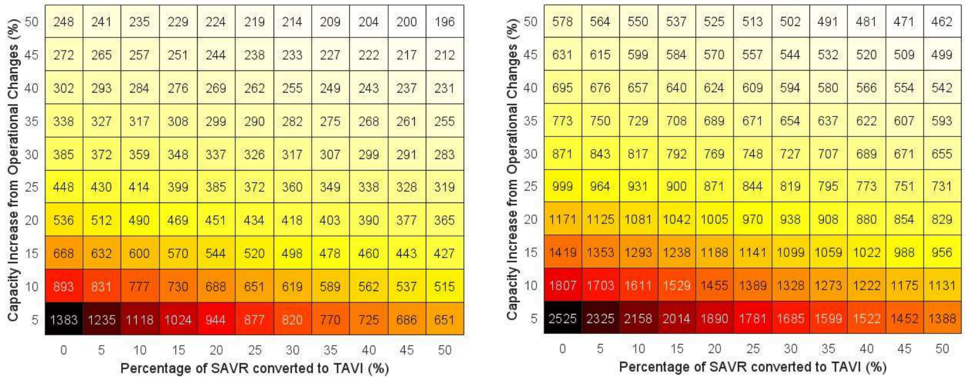

Supplementary Figure S1: Mean time to clear backlog (left) and the resulting deaths (right) as a function of daily percentage increase in capacity (y-axis) and percentage of SAVR converted to TAVI (x-axis), assuming that for every three SAVR operations, four TAVI procedures can be performed instead.

Supplementary figure S2: Heat map of a three-to-five SAVR-to-TAVI conversion

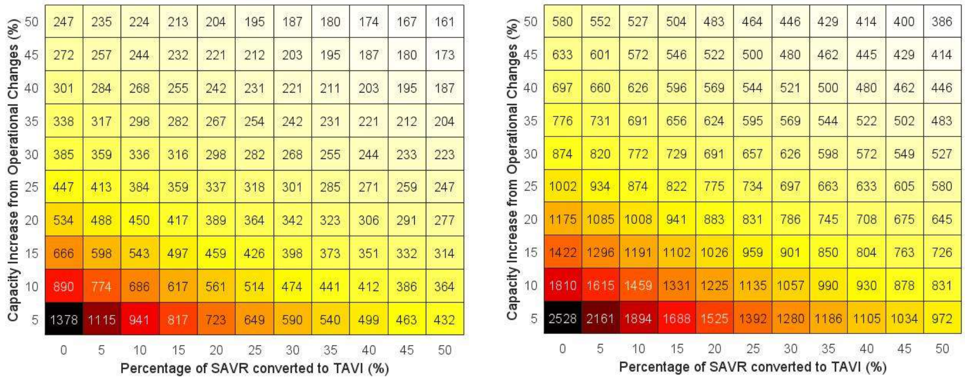

Supplementary Figure S2: Mean time to clear backlog (left) and the resulting deaths (right) as a function of daily percentage increase in capacity (y-axis) and percentage of SAVR converted to TAVI (x-axis), assuming that for every three SAVR operations, five TAVI procedures can be performed instead.

Supplementary figure S3: Heat map of a two-to-four SAVR-to-TAVI conversion

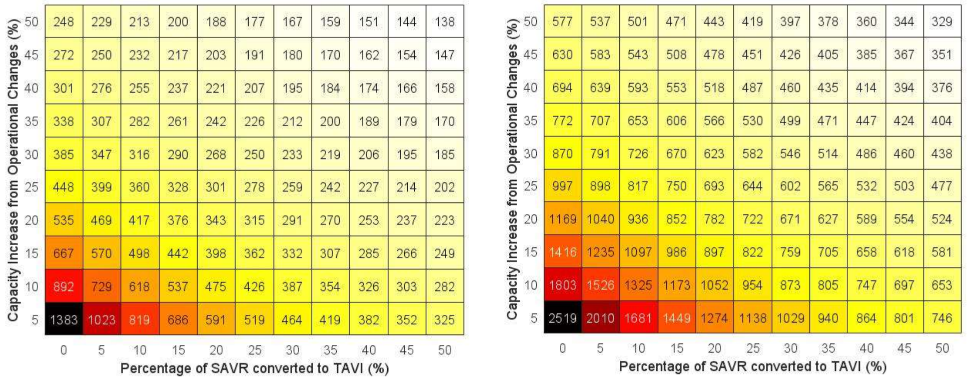

Supplementary Figure S3: Mean time to clear backlog (left) and the resulting deaths (right) as a function of daily percentage increase in capacity (y-axis) and percentage of SAVR converted to TAVI (x-axis), assuming that for every two SAVR operations, four TAVI procedures can be performed instead.

Supplementary figure S4: Error from mortality estimates

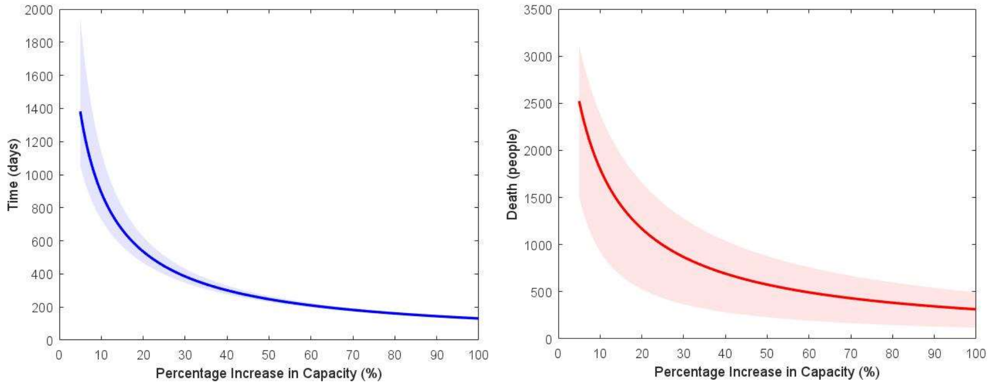

Supplementary figure S4: Time to clear backlog (left) and the resulting deaths (right) with associated 95% reference ranges as a function of daily percentage increase in capacity, with uncertainty from mortality only. The x-axis is truncated at 5% for visualisation and clarity.

We find that error in the one-year mortality causes higher uncertainty at lower capacity increases, but at higher capacity increases, this uncertainty decreases until it is almost zero with regards to clearance time. This is likely because at higher capacity increases, more of our waiting list clearance comes from treatment, as opposed to death, resulting in less error.

Supplementary figure S5: Error from wait list (W_0) estimates

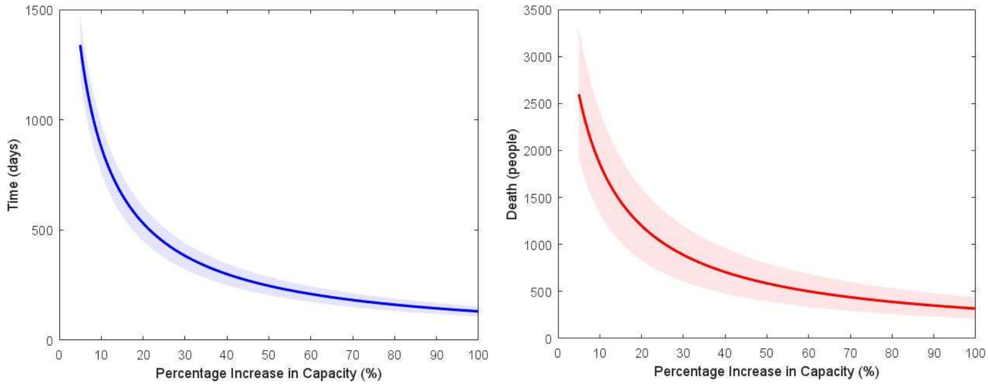

Supplementary figure S5: Time to clear backlog (left) and the resulting deaths (right) with associated 95% reference ranges as a function of daily percentage increase in capacity, with uncertainty from initial waiting list estimates only. The x-axis is truncated at 5% for visualisation and clarity.

We find that error in the estimate of the wait list length W_0 causes uncertainty that is fairly constant in the time it takes to clear the backlog and in resultant deaths. This is to be expected as we can show that the uncertainty scales with $\ln W_0$. There is a small decrease in uncertainty as we increase capacity, once again because an increase in capacity results in more control of the waiting list reduction.
