# Supplementary material for: Aortic stenosis post-COVID-19: a mathematical model on waiting lists and mortality
Source: BMJ Open. 2022 Jun 16;12(6):e059309. doi: 10.1136/bmjopen-2021-059309 (PMC9207579; doi:10.1136/bmjopen-2021-059309)
Supplement: Author's manuscript [file bmjopen-2021-059309.draft_revisions.pdf]

BMJ Open is committed to open peer review. As part of this commitment we make the peer review history of every article we publish publicly available.

When an article is published we post the peer reviewers' comments and the authors' responses online. We also post the versions of the paper that were used during peer review. These are the versions that the peer review comments apply to.

The versions of the paper that follow are the versions that were submitted during the peer review process. They are not the versions of record or the final published versions. They should not be cited or distributed as the published version of this manuscript.

BMJ Open is an open access journal and the full, final, typeset and author-corrected version of record of the manuscript is available on our site with no access controls, subscription charges or pay-per-view fees (http://bmjopen.bmj.com).

If you have any questions on BMJ Open's open peer review process please email info.bmjopen@bmj.com

BMJ Open

Aortic stenosis post-COVID-19: A mathematical model on waiting lists and mortality

Journal:	*BMJ Open*
Manuscript ID	bmjopen-2021-059309
Article Type:	Original research
Date Submitted by the Author:	23-Nov-2021
Complete List of Authors:	Stickels, Christian; University of Liverpool, Department of Mathematics<br>Nadarajah, Ramesh; University of Leeds, Leeds Institute for Data Analytics; University of Leeds, Leeds Institute of Cardiovascular and Metabolic Medicine<br>Gale, Chris; University of Leeds, Leeds Institute for Data Analytics; University of Leeds, Leeds Institute of Cardiovascular and Metabolic Medicine<br>Jiang, Houyuan; University of Cambridge, Judge Buisness School<br>Sharkey, Kieran J; University of Liverpool, Department of Mathematical Sciences<br>Gibbison, Ben; Bristol Medical School, Cardiac Anaesthesia and Intensive Care<br>Holliman, Nick; Newcastle University, School of Computing<br>Lombardo, Sara; Loughborough University, Mathematical Sciences<br>Schewe, Lars; The University of Edinburgh School of Mathematics<br>Sommacal, Matteo; Northumbria University, Department of Mathematics, Physics and Electrical Engineering<br>Sun, Louise; University of Ottawa Heart Institute, Division of Cardiac Anesthesiology; Institute for Clinical Evaluative Sciences,<br>Weir-McCall, Jonathan; University of Cambridge, Department of Radiology<br>Cheema, Katherine; British Heart Foundation<br>Rudd, James H F; University of Cambridge, Department of Radiology<br>Mamas, Mamas; Keele University, Keele Cardiovascular Research Group<br>Erhun, Feryal; University of Cambridge
Keywords:	Cardiology < INTERNAL MEDICINE, COVID-19, Valvular heart disease < CARDIOLOGY

SCHOLARONE™ Manuscripts

I, the Submitting Author has the right to grant and does grant on behalf of all authors of the Work (as defined in the below author licence), an exclusive licence and/or a non-exclusive licence for contributions from authors who are: i) UK Crown employees; ii) where BMJ has agreed a CC-BY licence shall apply, and/or iii) in accordance with the terms applicable for US Federal Government officers or employees acting as part of their official duties; on a worldwide, perpetual, irrevocable, royalty-free basis to BMJ Publishing Group Ltd ("BMJ") its licensees and where the relevant Journal is co-owned by BMJ to the co-owners of the Journal, to publish the Work in this journal and any other BMJ products and to exploit all rights, as set out in our licence.

The Submitting Author accepts and understands that any supply made under these terms is made by BMJ to the Submitting Author unless you are acting as an employee on behalf of your employer or a postgraduate student of an affiliated institution which is paying any applicable article publishing charge ("APC") for Open Access articles. Where the Submitting Author wishes to make the Work available on an Open Access basis (and intends to pay the relevant APC), the terms of reuse of such Open Access shall be governed by a Creative Commons licence – details of these licences and which Creative Commons licence will apply to this Work are set out in our licence referred to above.

Other than as permitted in any relevant BMJ Author's Self Archiving Policies, I confirm this Work has not been accepted for publication elsewhere, is not being considered for publication elsewhere and does not duplicate material already published. I confirm all authors consent to publication of this Work and authorise the granting of this licence.

For peer review only

Title

Aortic stenosis post-COVID-19: A mathematical model on waiting lists and mortality

Publication Type

Original Research

Authors

Christian P Stickels[1], Ramesh Nadarajah[2,3,4], Chris P Gale[2,3,4], Houyuan Jiang[5], Kieran J Sharkey[1], Ben Gibbison[6], Nick Holliman[7], Sara Lombardo[8], Lars Schewe[9], Matteo Sommacal[10], Louise Sun[11,12,13], Jonathan Weir-McCall[14], Katherine Cheema[15], James H F Rudd[16], Mamas A. Mamas[17], Feryal Erhun[5]

[1] Department of Mathematical Sciences, University of Liverpool, UK

[2] Leeds Institute for Cardiovascular and Metabolic Medicine, University of Leeds, UK

[3] Leeds Institute of Data Analytics, University of Leeds, UK

[4] Department of Cardiology, Leeds Teaching Hospitals NHS Trust, Leeds, UK

[5] Judge Business School, University of Cambridge, UK

[6] Cardiac Anaesthesia and Intensive Care, Bristol Medical School, University of Bristol, UK

[7] School of Computing, Newcastle University, UK

[8] Mathematical Sciences, Loughborough University, UK

[9] School of Mathematics, University of Edinburgh, UK

[10] Department of Mathematics, Physics and Electrical Engineering, Northumbria University, UK

[11] Division of Cardiac Anaesthesiology, University of Ottawa Heart Institute, Canada

[12] School of Epidemiology and Public Health, University of Ottawa, Canada

[13] Institute for Clinical Evaluative Sciences, Canada

[14] Department of Radiology, University of Cambridge, UK

[15] British Heart Foundation, UK

[16] Division of Cardiovascular Medicine, University of Cambridge, UK

[17] Keele Cardiovascular Research Group, Keele University, UK

Correspondence

Feryal Erhun

Judge Business School

For peer review only - http://bmjopen.bmj.com/site/about/guidelines.xhtml

University of Cambridge
Cambridge, UK
Email: f.erhun@jbs.cam.ac.uk

Word Count

2776

Keywords

Cardiology
COVID-19
Valvular heart disease

For peer review only

Abstract

Objectives

To provide estimates for how different treatment pathways for the management of severe aortic stenosis (AS) may affect NHS England waiting list duration and associated mortality.

Design

We constructed a mathematical model of the excess waiting list and found the closed-form analytic solution to that model. From published data, we calculated estimates for how the following strategies may affect the time to clear the backlog of patients waiting for treatment and the associated waiting list mortality.

Setting

The NHS in England.

Participants

Estimated aortic stenosis patients in England.

Interventions

1) increasing the capacity for the treatment of severe AS, 2) converting proportions of cases from surgery to transcatheter aortic valve implantation, and 3) a combination of these two.

Results

In a capacitated system, clearing the backlog by returning to pre-COVID-19 capacity is not possible. A conversion rate of 50% would clear the backlog within 666 (95% CI, 533–848) days with 1419 (95% CI, 597–2189) deaths whilst waiting during this time. A 20% capacity increase would require 535 (95% CI, 434–666) days, with an associated mortality of 1172 (95% CI, 466–1859). A combination of converting 40% cases and increasing capacity by 20% would clear the backlog within a year (343 (95% CI, 281–410) days) with 784 (95% CI, 292–1324) deaths whilst awaiting treatment.

For peer review only - http://bmjopen.bmj.com/site/about/guidelines.xhtml

Conclusion

A strategy change to the management of severe AS is required to reduce the NHS backlog and waiting list deaths during the post-COVID-19 'recovery' period. However, plausible adaptations will still incur a substantial wait and many hundreds dying without treatment.

For peer review only

For peer review only - http://bmjopen.bmj.com/site/about/guidelines.xhtml

What is already known on this subject?

It has been estimated that almost 5000 patients were left untreated for severe aortic stenosis in England due to indirect effects of the COVID-19 pandemic up to November 2020. However, to our knowledge, there has been no published literature examining how to manage the extra backlog this will cause on the waiting list for treatment.

What might this study add?

In this study, we found that without significant intervention, the waiting list of patients seeking treatment for severe aortic stenosis will not return to pre-pandemic levels for several years, resulting in thousands of preventable deaths. This study presents a model for evaluating the relative efficacy of different interventions, including adding extra treatment capacity and converting a proportion of cases to TAVI from surgery, to clear the backlog and minimise mortality of patients waiting for treatment.

Strengths and limitations of this study

- This model's greatest strength is that it provides a good basis to begin to solve a time-critical problem when data gathering is likely to result in a greater number of deaths.
- The discussion about how treating some SAVR patients with TAVI instead is a useful tool that examines how giving some patients what might be seen by some as sub-optimal treatment, results in better overall outcomes for the target population.
- The assumption that the entire NHS can be modelled as a single entity with a single waiting list is a limitation of this study.
- We also recognise that the waiting numbers we use in our study are likely to be flawed as we do not know how many AS patients have died due to catching COVID-19.

For peer review only - http://bmjopen.bmj.com/site/about/guidelines.xhtml

Introduction

The COVID-19 pandemic has led to the reorganisation of healthcare services to limit the transmission of the virus and deal with the sequelae of infection. This reorganisation had a detrimental effect on cardiovascular services, with reductions in hospitalisations for acute cardiovascular events and the deferral of all but the most urgent interventional procedures and operations.[1, 2]

Aortic stenosis (AS) is the most common form of valvular heart disease. Once stenosis is severe, symptoms follow and the prognosis is poor, with 50% mortality within two years of symptom onset.[3] Thus, timely treatment is of paramount importance. Surgical aortic valve replacement (SAVR) has historically been the default treatment strategy. However, transcatheter aortic valve implantation (TAVI) has recently emerged as an effective and increasingly utilised option across operative risk strata.[4-8]

There was a large decline in TAVI and SAVR procedural activity to treat severe AS during the COVID-19 pandemic.[9] Between the period March to November 2020, it is estimated that the decrease in activity accounted for 4989 (95% CI. 4020–5959) patients in England with severe AS left untreated by TAVI or SAVR.[9] As we move into an era of ‘living with’ COVID-19, plans must urgently be put in place to best manage the additional waiting list burden for treatment of severe AS.[10]

In this study, we used mathematical methods to examine the extent to which additional capacity to provide treatment of severe AS should be created to clear the backlog and minimise deaths of people on the waiting list.

Methods

Study population and assumptions

Data from the UK TAVR registry and NICOR (National Institute for Cardiovascular Outcomes Research) National Adult Cardiac Surgery Audit (NACSA) between 2017 and 2020 have previously been extracted to estimate an excess waiting list size (W_0) of 4989

For peer review only - http://bmjopen.bmj.com/site/about/guidelines.xhtml

(95% CI, 4020–5959) patients with severe AS left untreated as of November 2020.[9] In the absence of contemporaneous data on waiting lists and SAVR and TAVI activity, we have taken this number as the excess backlog on which to model solutions. The incidence of AS has not increased over recent years.[11] Therefore, we assumed that the system was in a steady state before the COVID-19 pandemic and without loss of generality defined the steady-state waiting list to be zero. Additionally, we assumed that the normal rate of flow (f) of new patients into the waiting list for treatment of severe AS would be maintained upon the commencement of additional operations. Thus, the extra capacity that we model is to clear the excess post-COVID-19 backlog.

We took one-year mortality (μ) after the onset of symptoms in severe AS to be 36% (95% CI, 12% – 60%).[12] More recent studies have estimated the one-year mortality to be 51%[5] and 55%, but these included cohorts that were considered inappropriate for SAVR, thus, we considered these estimates unrepresentative of an unselected population with severe AS.[13] The routine capacity for treatment of severe AS was taken from the pre-pandemic period. In 2018/19, the NHS in England performed 7830 SAVR ($r_S^0 = 7830$) and 5197 TAVI ($r_T^0 = 5197$) procedures, for a total throughput of about 13,000 per year.[14]

Modelling

Patients on the waiting list for treatment of severe AS were represented as a dynamical system (figure 1).

To this model, we introduce capacity in surplus to the 2018/19 performance and call this capacity T_e (further details are provided in supplementary material). We assume that the typical caseload for which the NHS in England can deal with continues; therefore, the backlog is only reduced by treating patients with this extra capacity or by patient mortality before receiving treatment. We also consider patients in the backlog and patients new to the waiting list indistinguishable. Thus, the waiting list size represents the excess number of people seeking treatment who are unable to be treated immediately at any one time. We also assume that other paths out of the waiting list (i.e. patients seeking private treatment) would be so small in comparison to the uncertainty estimates as to be negligible on the results of our analysis.

For peer review only - http://bmjopen.bmj.com/site/about/guidelines.xhtml

These assumptions then come together to give an estimated time (see supplementary material for derivation) to clear the waiting list (t_C)

$$t_C = \frac{ln\left(1 + \frac{W_0\mu}{T_e}\right)}{\mu} \quad (1)$$

and associated mortality ($m(t_C)$)

$$m(t_C) = W_0 - T_e t_C. \quad (2)$$

Using equations (1) and (2), we can predict the length of time and associated mortality for different percentage increases in capacity. We assume any capacity increase to be constant throughout the entire modelled period. For example, if we increased daily capacity by 5% this would result in, $T_e = \frac{r_S^0 + r_T^0}{365} * 5\% = 1.785$ extra procedures per day, across the whole of the NHS in England. We generated 10,000 random values for the one-year mortality rate and initial waiting list length. We assumed that the uncertainty in both variables was normally distributed.

Interventions and outcomes

We investigated three types of capacity increase: 1) a general increase in the capacity to provide SAVR and TAVI, which could be facilitated by an increased number of procedures per list, additional lists, and prioritisation of care pathways and staffing to treat severe AS; 2) extra capacity created by treating some patients with TAVI who would routinely have SAVR; 3) a combination of a general increase in capacity and the conversion of a proportion of cases from SAVR to TAVI. During the COVID-19 pandemic, TAVI was performed in patients usually referred for surgery, with no difference in short term outcomes compared to historical reference groups.[15, 16]

We assumed that the duration of a SAVR would routinely be between 2-4 hours and a TAVI between 1-2 hours.[17, 18] As such, we assumed within the time for two SAVR operations, three TAVI could be performed instead.[19] Several clinical factors may favour SAVR over TAVI (including concomitant severe coronary artery disease, low STS score, bicuspid aortic valve etc.); therefore, we assumed that, in the short term, no more than 50% of patients could be converted from SAVR to TAVI.[20] We also assumed that no more than 50% extra

For peer review only - http://bmjopen.bmj.com/site/about/guidelines.xhtml

capacity could be created by other means (e.g. extra lists, more procedures per list). We simulated two principal outcomes based on the creation of additional capacity (T_e):

1. Time to clear the backlog (reduce to zero),
2. Mortality of patients within the excess backlog whilst on the waiting list to be treated.

We completed additional sensitivity analyses for how the conversion of SAVR to TAVI would affect the principal outcomes, including if three SAVR operations could be routinely completed in a day and four to five TAVI procedures per day (presuming increasing uptake of a minimalist TAVI approach without general anaesthesia enabling more rapid procedure time).[21]

Patient and public involvement

Patients and the public were not involved in the conduct of this study.

Results

In the pre-COVID-19 period, the routine capacity for treatment of severe AS was set to cover the normal incident rate. That is, clearing the backlog by returning to pre-COVID-19 capacity is not possible. As a result, mortality on the excess waiting list at one year are estimated to be more than 1500, putting a strong emphasis on the need for change.

Total additional capacity

Figure 2 provides simulations of the time to clear the excess backlog and the mortality of patients on the waiting list based on the amount of total additional capacity, T_e. With a 5% increase in the capacity to provide treatment of severe AS, we estimate it would take 1384 (95% CI, 1025–1994) days to clear the excess backlog, with 2526 (95% CI, 1355–3516) deaths. A 20% increase in total capacity would provide a sharp benefit in clearing the excess backlog within 536 (95% CI, 434–666) days, with an estimate of 1173 (95% CI, 466–1859) deaths. As total capacity increases further, there is a diminishing return in clearing the backlog and avoiding associated mortality; the greater the capacity increase, the fewer lives are saved for every extra increase in capacity. Even if it was possible to double capacity, it

For peer review only - http://bmjopen.bmj.com/site/about/guidelines.xhtml

would take 131 (95% CI, 126–137) days to clear the backlog and there would be 313 (95% CI, 118–494) deaths on the waiting list.

The effect of converting SAVR to TAVI

The conversion of a proportion of cases from surgery to TAVI provides a modest improvement in estimates of time to clear the backlog and mortality on the waiting list. With the conversion of 30% of SAVR operations to TAVI procedures, without the creation of additional capacity in the system, we estimate it would take 975 (95% CI, 741–1284) days to clear the backlog and result in 1914 (95% CI, 923–2809) deaths on the waiting list. Even with the conversion of 50% of SAVR operations to TAVI procedures, we estimate the backlog would be cleared within 666 (95% CI, 533–848) days with 1419 (95% CI, 597–2189) deaths.

Combining conversion of SAVR to TAVI and additional capacity

Figures 3a and 3b demonstrate the range of possibilities in creating extra capacity. Each line demonstrates a range of intervention strategies that provide the same result. For example, to reduce mean predicted deaths to 1000 people (red line figure 3b), centres could increase capacity to provide an extra 25% procedures per week at the same mix as pre-pandemic, or they could convert 50% of SAVR operations to TAVI and increase capacity by 8.7% at that mix. Figures 3c and 3d represent how the combinations of interventions to increase capacity within the system alongside the conversion of SAVR to TAVI would impact the time to clear the backlog and on the associated mortality of waiting. Mortality on the waiting list is less responsive to our modelled interventions than the time to clear the backlog (the darker coloured regions of figure 3d make up a greater proportion of the estimates than those of figure 3c). Increasing capacity within the system alongside converting a proportion of SAVR cases to TAVI provides the greatest benefit in clearing the backlog and avoiding associated mortality. A combination that would result in the clearance of the backlog within a year might be of interest for decision makers. With the conversion of 40% of SAVR operations to TAVI and creation of an additional 20% capacity, we estimate the backlog would be cleared in just under a year – 343 days (95% CI, 281–410) with 784 (95% CI, 292–1324) deaths before treatment. Sensitivity analyses where the number of TAVI procedures that could be completed within the same time as SAVR was altered (TAVI to SAVR: 4 to 3, 4 to 2, 5 to 3) support these findings (supplementary material figures S1 – S3). Furthermore, sensitivity analyses show that with the best-in-class practices (TAVI to SAVR: 4 to 2), even a more

For peer review only - http://bmjopen.bmj.com/site/about/guidelines.xhtml

modest combination (a conversion of 35% and creation of an additional 10% capacity) would be enough to clear the backlog within a year.

Discussion

In this study, using dynamical system modelling, we provide estimates for how changes to treatment pathways for severe aortic stenosis may affect the time taken to clear the backlog and minimise mortality on the waiting list in the NHS of England. Without providing at least 20% total additional capacity for the interventional treatment of AS, we estimated there would be more than 1000 deaths on the waiting list over a period of nearly 1.5 years. A conversion of cases from SAVR to TAVI would expedite the clearance of the backlog, but even converting half the cases to TAVI would still result in over 1400 deaths over a period of almost 2 years. A combination of converting 40% of cases usually planned for SAVR to TAVI and creating 20% additional capacity for procedures (through measures such as extra lists) would clear the excess backlog within one year, with 784 deaths.

Our study has several strengths. First, in an urgent situation of many unknowns, our use of novel mathematical models provides plausible estimates on which to base planning and provides an exemplar that may be used in service delivery in other conditions in the post-pandemic landscape. Given the high event rate amongst this population, waiting for more contemporary data to be collected may well not provide enough time to institute system changes to prevent deaths. Second, we also provide specific estimates for how the conversion of cases to TAVI from surgery may affect waiting lists and associated mortality, which can inform local MDT discussions. Third, our model can act as a basis for a clinical and cost-benefit analysis to evaluate different ways to increase capacity and define the optimal strategy at each centre. For each centre, the most effective combination of converting SAVR to TAVI and provision or prioritisation of treatment of severe AS can be generated.

We also recognise the limitations inherent in modelling a complex situation. First, we represent the NHS in England as a single entity. As such, we implicitly assume that population and capacity are distributed evenly throughout the country by treating centre capacity. If the distribution of waiting list patients deviates significantly from the distribution

For peer review only - http://bmjopen.bmj.com/site/about/guidelines.xhtml

of treatment centres weighted by capacity, the time it would take to clear the waiting list, and thus the mortality rate would be higher. Second, we have not attempted to calculate how many AS patients may have died in the COVID-19 pandemic. Third, our assumed mortality rate may differ at a centre-level due to prioritising clinically more vulnerable patients on the waiting list. Fourth, a centre-level analysis could account for the different practices in each treatment centre and identify strategies that work best for each centre. Fifth, our estimates from converting cases from SAVR to TAVI does not include post-procedural factors such as the requirement for intensive care capacity, hospital stay and further procedures because these rely on multiple centre-specific factors. Finally, it has been shown that rapid growth in the demand for TAVI can overwhelm current capacity,[22] which may lead to prolonged wait times and subsequent adverse outcomes while patients are on the waitlist. Therefore, a demand model that captures the changes of demand for TAVI and SAVR would be a helpful future direction of analysis.

A previous study used a mathematical model to quantify the cumulative cardiac surgical backlog (including coronary artery bypass grafting surgery, valve replacement and transcatheter aortic and mitral valve replacements) in two centres based on the projected pandemic duration in the United States of America (USA).[23] The authors used simple mathematical models to predict the time required to clear the backlog depending on increased operating capacity. However, the authors did not consider mortality, which we have as it is of critical importance to patients and when planning services.

The results of our study highlight concerns pertaining to the deferral of non-emergency treatment for severe AS during the ‘recovery period’ of COVID-19. Severe AS is a progressive condition with valve replacement the only available treatment improving prognosis.[24] On a local, regional, and national scale, healthcare systems will need to examine capacity, set priorities, and plan for adequate capacity to manage the backlog of patients with severe AS. The response will be complicated by prior exhaustion of human resources from the pandemic and competition with other specialities, which will also have backlogs.[25]

Nonetheless, planning should prioritise patients at the highest risk from a deferral of treatment. Mortality on the waiting list for AS has been reported to be as high as 14%.[26]

For peer review only - http://bmjopen.bmj.com/site/about/guidelines.xhtml

Furthermore, patients awaiting structural procedures deferred due to the pandemic have been found to have significantly higher mortality rates compared to those with stable coronary artery disease.[27] Prioritising capacity for treatment of patients with severe AS may mean reduced capacity for other procedures. This interaction will require collaborative decision-making on a local level accepting that these are difficult, imperfect times. We also show that the conversion of a proportion of cases that would usually be managed by SAVR to TAVI can help expedite treatment and reduce mortality on the waiting list. During the pandemic, TAVI procedures were performed in patients usually referred for surgery with no apparent difference in short term outcomes;[15, 16] and data continues to emerge for longer-term efficacy and safety of TAVI across operative risk strata.[28,29] Recent European guidelines suggest that TAVI would be a preferable option for patients over 75 years of age compared to SAVR.[20] To help planning, we provide an app (https://github.com/Christian-P-Stickels/AS_Waitinglist_data) to explore the impact of alterations in capacity and treatment pathways on waiting lists and mortality related to severe AS at a local, regional and national level (supplementary material).

Conclusions

In this study, we identify that without a combination of increased capacity for treatment of patients with severe aortic stenosis, and consideration of expanding the use of TAVI, there will be unpalatable rates of mortality in this high-risk group during the post-COVID-19 ‘recovery’ period. These results should inform the planning of cardiac services.

Acknowledgement

We want to thank all the participants of the V-KEMS Study Group on “Modelling Solutions to the Impact of COVID-19 on Cardiovascular Waiting Lists” that took place on February 2-4, 2021, for thought-provoking discussions. Our special thanks to Clare Merritt (Newton Gateway to Mathematics), whose help extended beyond the workshop and was crucial in completing this work, and to Alan Champneys who brought the group together in the first place. BG is supported by the NIHR Bristol Biomedical Research Centre at the University of Bristol and

For peer review only - http://bmjopen.bmj.com/site/about/guidelines.xhtml

University Hospitals Bristol and Weston NHS Foundation Trust. JHFR is part-supported by the NIHR Cambridge Biomedical Research Centre, the British Heart Foundation, HEFCE, the EPSRC Cambridge Centre for Mathematics of Information in Healthcare and the Wellcome Trust.

Funding

This study was part funded by EPSRC Cambridge Centre for Mathematics of Information in Healthcare, grant number EP/T017961/1. Further, this study has in-principle funding from the Newton Institute, but at time of submission, this has not been approved. None of these funding sources had an impact on the design, data analysis, writing of, or decision to publish this paper.

Competing Interests

CG acknowledges grants not related to this project from Abbott, the British Heart Foundation, and Deputy Editorship at EuroHeart. BG acknowledges grants not related to this project from the David Telling Charitable Trust, and the Biotechnology and Biological Sciences Research Council, he additionally declared Associate Editorship of Anesthesia Journal, and being the chair DMSC for the COPIA Trial. All other authors confirm that they have no competing interests to declare.

Data Sharing

No additional data available

Contributorship statement

MM proposed the initial workshop, MM, CG, RN, BG and JHFR all helped to run said workshop as clinical experts. All members but KC and FE were involved in conceptualisation in the initial workshop. CS, HJ, KS, and FE designed the model with clinical guidance from MM, CG, RN, BG and JHFR. CS performed data analysis. CS, RJ and FE wrote the initial manuscript. All authors helped to improve the final manuscript. All authors approved the final manuscript.

Ethics Statement

This paper did not require ethics approval.

For peer review only - http://bmjopen.bmj.com/site/about/guidelines.xhtml

References

[1] Mohamed MO, Banerjee A, Clarke S, *et al.* Impact of COVID-19 on cardiac procedure activity in England and associated 30-day mortality. *European Heart Journal - Quality of Care and Clinical Outcomes* 2021;**7**:247–56. doi:10.1093/ehjqcco/qcaa079

[2] Bhatt AS, Moscone A, McElrath EE, *et al.* Fewer Hospitalizations for Acute Cardiovascular Conditions During the COVID-19 Pandemic. *Journal of the American College of Cardiology* 2020;**76**:280–8. doi:10.1016/j.jacc.2020.05.038

[3] Généreux P, Stone GW, O'Gara PT, *et al.* Natural History, Diagnostic Approaches, and Therapeutic Strategies for Patients with Asymptomatic Severe Aortic Stenosis. *Journal of the American College of Cardiology* 2016;**67**:2263–88. doi:10.1016/j.jacc.2016.02.057

[4] Leon MB, Smith CR, Mack M, *et al.* Transcatheter Aortic-Valve Implantation for Aortic Stenosis in Patients Who Cannot Undergo Surgery. *New England Journal of Medicine* 2010;**363**:1597–607. doi:10.1056/NEJMoa1008232

[5] Leon MB, Smith CR, Mack MJ, *et al.* Transcatheter or Surgical Aortic-Valve Replacement in Intermediate-Risk Patients. *New England Journal of Medicine* 2016;**374**:1609–20. doi:10.1056/NEJMoa1514616

[6] Smith CR, Svensson LG, Makkar RR, *et al.* Transcatheter versus Surgical Aortic-Valve Replacement in High-Risk Patients. *The New England Journal of Medicine* 2011;:12. doi:10.1056/NEJMoa1103510

[7] Mack MJ, Leon MB, Thourani VH, *et al.* Transcatheter Aortic-Valve Replacement with a Balloon-Expandable Valve in Low-Risk Patients. *New England Journal of Medicine* 2019;**380**:1695–705. doi:10.1056/NEJMoa1814052

[8] Popma JJ, Deeb GM, Yakubov SJ, *et al.* Transcatheter Aortic-Valve Replacement with a Self-Expanding Valve in Low-Risk Patients. *New England Journal of Medicine* 2019;**380**:1706–15. doi:10.1056/NEJMoa1816885

For peer review only - http://bmjopen.bmj.com/site/about/guidelines.xhtml

[9] Martin GP, Curzen N, Goodwin AT, *et al.* Indirect Impact of the COVID-19 Pandemic on Activity and Outcomes of Transcatheter and Surgical Treatment of Aortic Stenosis in England. *Circ: Cardiovascular Interventions* 2021;**14**. doi:10.1161/CIRCINTERVENTIONS.120.010413

[10] Ro R, Khera S, Tang GHL, *et al.* Characteristics and Outcomes of Patients Deferred for Transcatheter Aortic Valve Replacement Because of COVID-19. *JAMA Netw Open* 2020;**3**:e2019801. doi:10.1001/jamanetworkopen.2020.19801

[11] Martinsson A, Li X, Andersson C, *et al.* Temporal Trends in the Incidence and Prognosis of Aortic Stenosis: A Nationwide Study of the Swedish Population. *Circulation* 2015;**131**:988–94. doi:10.1161/CIRCULATIONAHA.114.012906

[12] Schwarz F, Baumann P, Manthey J, *et al.* The effect of aortic valve replacement on survival. *Circulation* 1982;**66**:1105–10. doi:10.1161/01.CIR.66.5.1105

[13] Wenaweser P, Pilgrim T, Kadner A, *et al.* Clinical Outcomes of Patients With Severe Aortic Stenosis at Increased Surgical Risk According to Treatment Modality. *Journal of the American College of Cardiology* 2011;**58**:2151–62. doi:10.1016/j.jacc.2011.05.063

[14] NICOR. National Adult Cardiac Surgery Report, 2020 Summary Report. https://www.nicor.org.uk/national-cardiac-audit-programme/adult-cardiac-surgery-surgery-audit/ (Accessed 17 September 2021)

[15] Adlam D, Chan N, Baron J, *et al.* Aortic stenosis in the time of COVID-19: Development and outcomes of a rapid turnaround TAVI service. *Catheter Cardiovasc Interv* 2021;**98**. doi:10.1002/ccd.29550

[16] Shafi AMA, Awad WI. Transcatheter aortic valve implantation versus surgical aortic valve replacement during the COVID-19 pandemic—Current practice and concerns. *J Card Surg* 2021;**36**:260–4. doi:10.1111/jocs.15182

For peer review only - http://bmjopen.bmj.com/site/about/guidelines.xhtml

[17] Aortic valve replacement. nhs.uk. 2017. https://www.nhs.uk/conditions/aortic-valve-replacement/ (accessed 13 Oct 2021).

[18] TAVI (Transcatheter aortic valve implantation). https://www.bhf.org.uk/informationsupport/treatments/tavi (accessed 13 Oct 2021).

[19] Martin JA, Mayhew CR, Morris AJ, *et al.* Using Time-Driven Activity-Based Costing as a Key Component of the Value Platform: A Pilot Analysis of Colonoscopy, Aortic Valve Replacement and Carpal Tunnel Release Procedures. *J Clin Med Res* 2018;**10**:314–20. doi:10.14740/jocmr.v10i4.3350

[20] Vahanian A, Beyersdorf F, Praz F, *et al.* 2021 ESC/EACTS Guidelines for the management of valvular heart disease. *European Heart Journal* 2021;:ehab395. doi:10.1093/eurheartj/ehab395

[21] Ali N, Faour A, Rawlins J, *et al.* 'Valve for Life': tackling the deficit in transcatheter treatment of heart valve disease in the UK. *Open Heart* 2021;**8**:e001547. doi:10.1136/openhrt-2020-001547

[22] Henning KA, Ravindran M, Qiu F, *et al.* Impact of procedural capacity on transcatheter aortic valve replacement wait times and outcomes: a study of regional variation in Ontario, Canada. *Open Heart* 2020;**7**:e001241. doi: 10.1136/openhrt-2020-001241

[23] Salenger R, Etchill EW, Ad N, *et al.* The Surge After the Surge: Cardiac Surgery Post–COVID-19. *The Annals of Thoracic Surgery* 2020;**110**:2020–5. doi:10.1016/j.athoracsur.2020.04.018

[24] Khialani B, MacCarthy P. Transcatheter management of severe aortic stenosis during the COVID-19 pandemic. *Heart* 2020;**106**:1183–90. doi:10.1136/heartjnl-2020-317221

[25] Perdoncin E, Greenbaum AB, Grubb KJ, *et al.* Safety of same-day discharge after uncomplicated, minimalist transcatheter aortic valve replacement in the COVID-19 era. *Catheter Cardiovasc Interv* 2021;**97**:940–7. doi:10.1002/ccd.29453

For peer review only

For peer review only - http://bmjopen.bmj.com/site/about/guidelines.xhtml

[26] Nuis RJ, Dager AE, van der Boon RM, *et al.* Patients with aortic stenosis referred for TAVI: treatment decision, in-hospital outcome and determinants of survival. *Neth Heart J* 2012;**20**:16–23. doi:10.1007/s12471-011-0224-z

[27] Moreno R, Díez J, Diarte J, *et al.* Consequences of canceling elective invasive cardiac procedures during Covid-19 outbreak. *Catheter Cardiovasc Interv* 2021;**97**:927–37. doi:10.1002/ccd.29433

[28] Blackman DJ, Saraf S, MacCarthy PA, *et al.* Long-Term Durability of Transcatheter Aortic Valve Prostheses. *Journal of the American College of Cardiology* 2019;**73**:537–45. doi:10.1016/j.jacc.2018.10.078

[29] Jørgensen TH, Thyregod HGH, Ihlemann N, *et al.* Eight-year outcomes for patients with aortic valve stenosis at low surgical risk randomized to transcatheter vs. surgical aortic valve replacement. *European Heart Journal* 2021;**42**:2912–9. doi:10.1093/eurheartj/ehab375

For peer review only

For peer review only - http://bmjopen.bmj.com/site/about/guidelines.xhtml

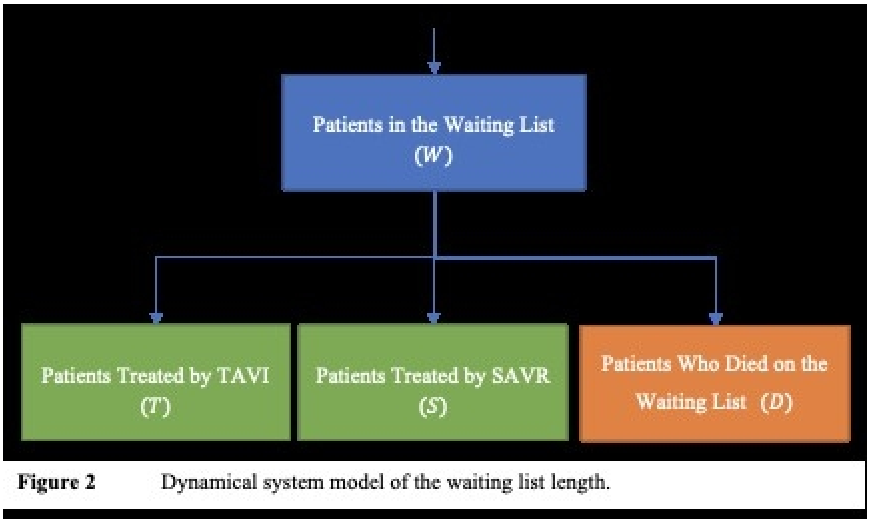

Figure 1: Dynamical system model of the waiting list length

114x67mm (96 x 96 DPI)

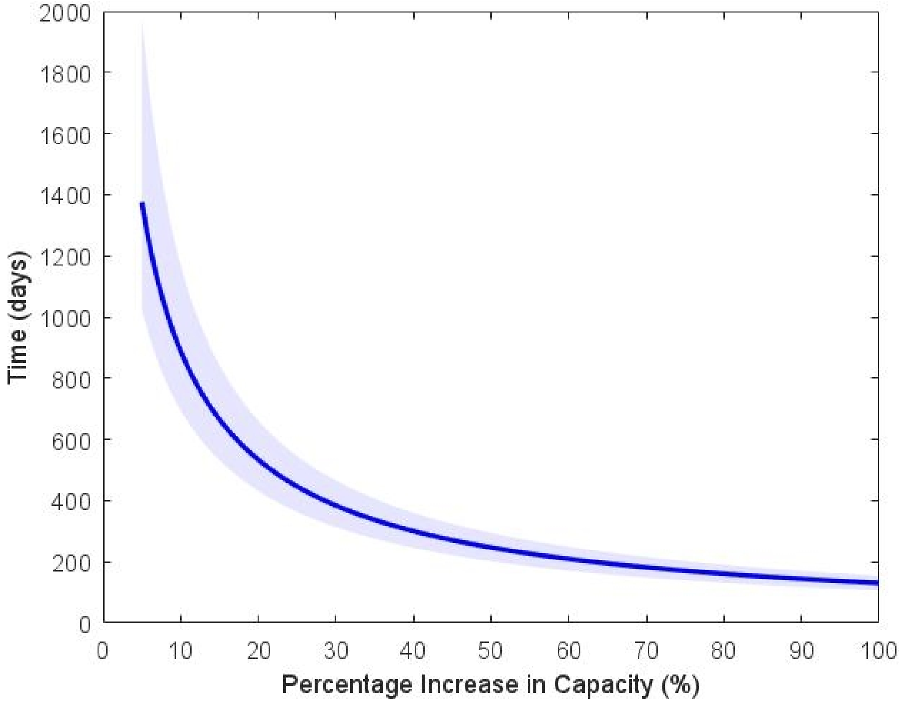

Figure 2a: Time to clear backlog (left) and the resulting deaths (right) with associated 95% confidence intervals as a function of daily percentage increase in capacity, with uncertainty from mortality and the initial waiting list. The x-axis is truncated at 5% for visualisation and clarity.

246x185mm (72 x 72 DPI)

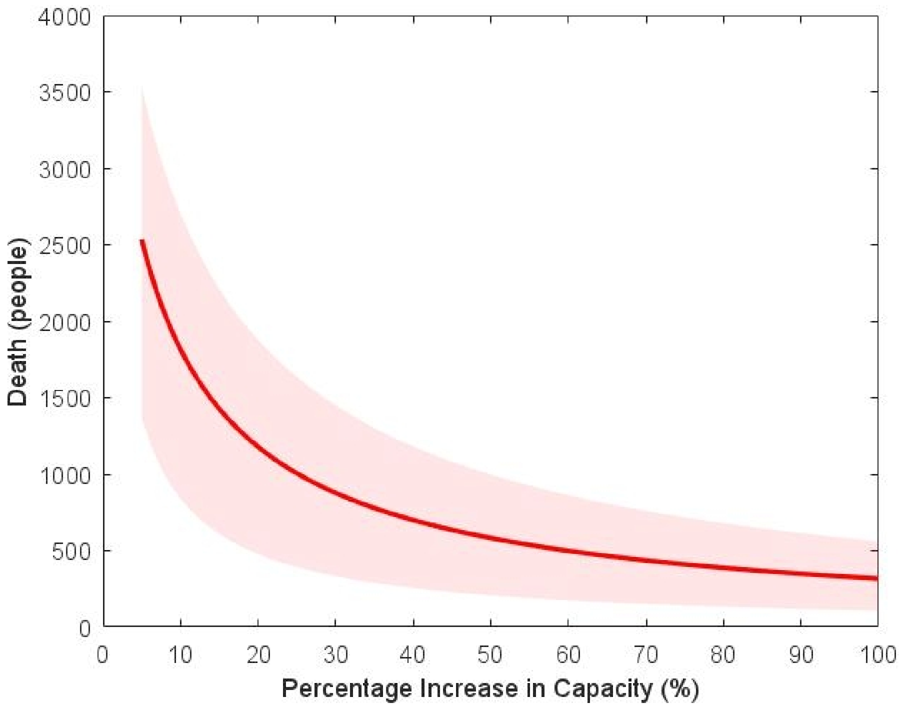

Figure 2b: Time to clear backlog (left) and the resulting deaths (right) with associated 95% confidence intervals as a function of daily percentage increase in capacity, with uncertainty from mortality and the initial waiting list. The x-axis is truncated at 5% for visualisation and clarity.

246x185mm (72 x 72 DPI)

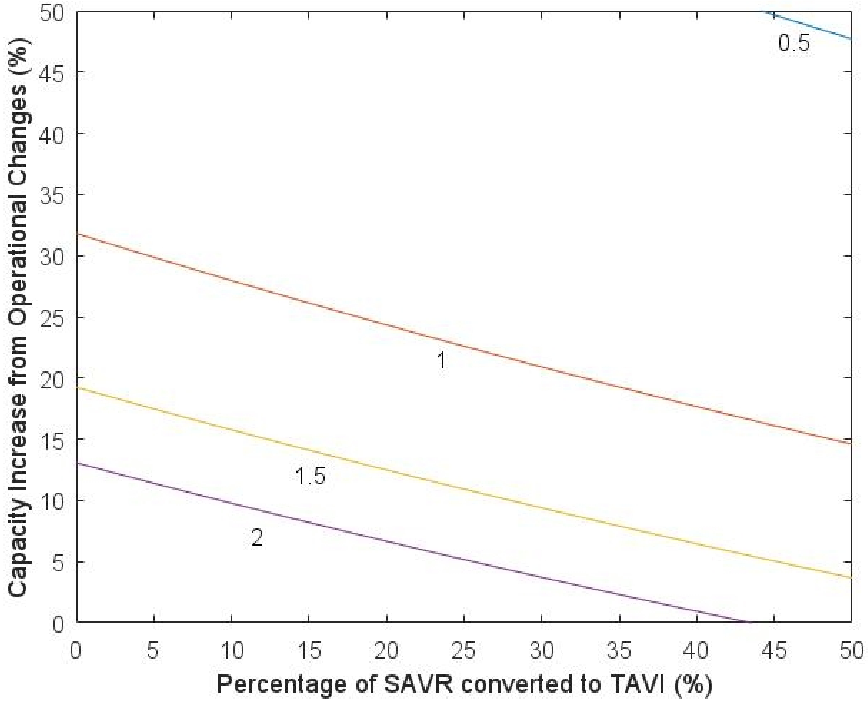

Figure 3a: Mean time to clear backlog (left) and the resulting deaths (right) as a function of daily percentage increase in capacity (y-axis) and percentage of SAVR converted to TAVI (x-axis) (Presented in two different forms). A) Isoclines of constant mean clearance-time going from half a year (blue) to 2 years (purple) in half-year increments. B) Isoclines of constant mean mortality after clearing the backlog from 500 people (blue) to 2000 (purple) in 500-person increments. C) Heatmap of different combinations of conversion and daily capacity increases and how long the backlog would take to clear on average, in days. D) Heatmap of different combinations of conversion and daily capacity increases and how many people would die, on average.

246x185mm (72 x 72 DPI)

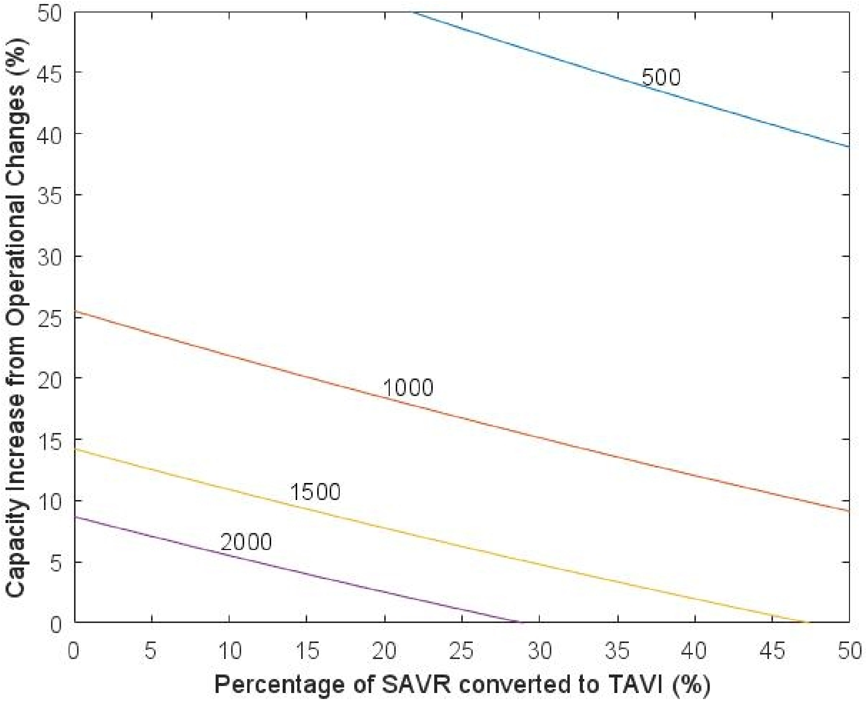

Figure 3b: Mean time to clear backlog (left) and the resulting deaths (right) as a function of daily percentage increase in capacity (y-axis) and percentage of SAVR converted to TAVI (x-axis) (Presented in two different forms). A) Isoclines of constant mean clearance-time going from half a year (blue) to 2 years (purple) in half-year increments. B) Isoclines of constant mean mortality after clearing the backlog from 500 people (blue) to 2000 (purple) in 500-person increments. C) Heatmap of different combinations of conversion and daily capacity increases and how long the backlog would take to clear on average, in days. D) Heatmap of different combinations of conversion and daily capacity increases and how many people would die, on average.

246x185mm (72 x 72 DPI)

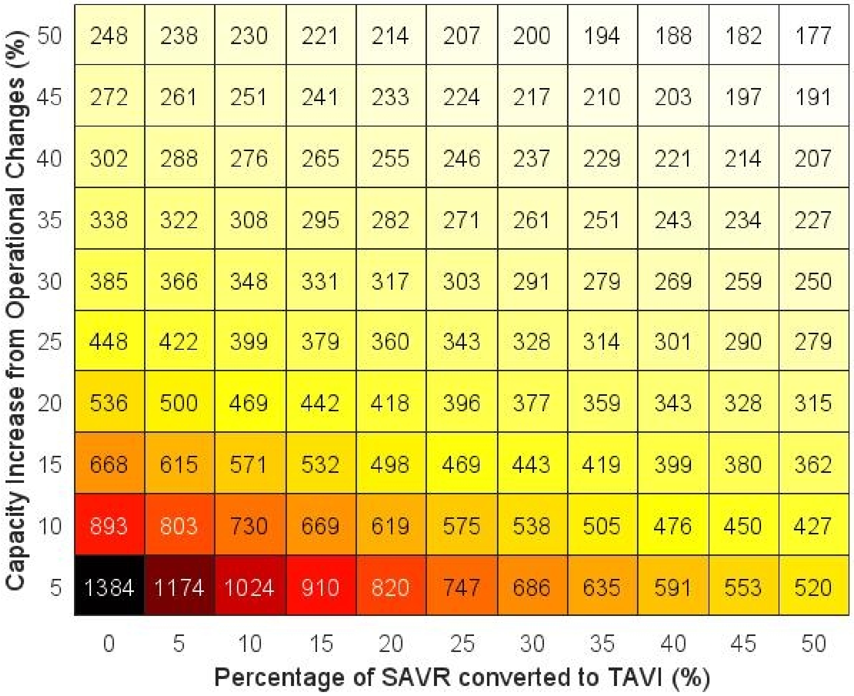

Figure 3c: Mean time to clear backlog (left) and the resulting deaths (right) as a function of daily percentage increase in capacity (y-axis) and percentage of SAVR converted to TAVI (x-axis) (Presented in two different forms). A) Isoclines of constant mean clearance-time going from half a year (blue) to 2 years (purple) in half-year increments. B) Isoclines of constant mean mortality after clearing the backlog from 500 people (blue) to 2000 (purple) in 500-person increments. C) Heatmap of different combinations of conversion and daily capacity increases and how long the backlog would take to clear on average, in days. D) Heatmap of different combinations of conversion and daily capacity increases and how many people would die, on average.

246x185mm (72 x 72 DPI)

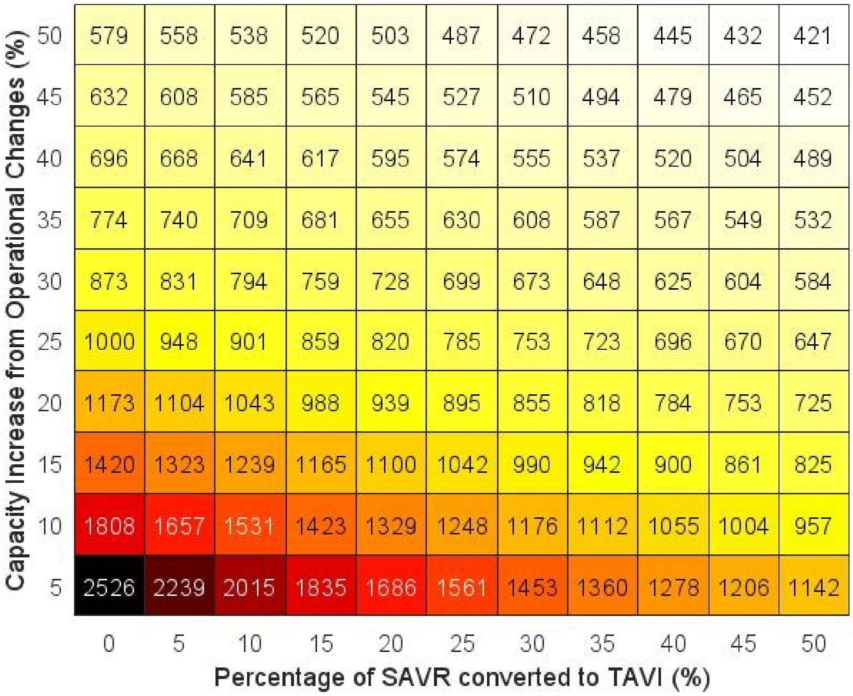

Figure 3d: Mean time to clear backlog (left) and the resulting deaths (right) as a function of daily percentage increase in capacity (y-axis) and percentage of SAVR converted to TAVI (x-axis) (Presented in two different forms). A) Isoclines of constant mean clearance-time going from half a year (blue) to 2 years (purple) in half-year increments. B) Isoclines of constant mean mortality after clearing the backlog from 500 people (blue) to 2000 (purple) in 500-person increments. C) Heatmap of different combinations of conversion and daily capacity increases and how long the backlog would take to clear on average, in days. D) Heatmap of different combinations of conversion and daily capacity increases and how many people would die, on average.

246x185mm (72 x 72 DPI)

SUPPLEMENTS

Supplement 1: Mathematical Derivation of the Differential Equation and its Solution

From figure 1, we can write the following equation:

$$\frac{dW}{dt} = f - r_T - r_S - \mu W.$$

We can then re-write and integrate this equation

$$\int_0^{t_c} 1dt = \int_{W_0}^{0} \frac{1}{f - r_T - r_S - \mu W} dW$$

$$t_c = \left[-\frac{1}{\mu} ln\,(f - r_T - r_s - \mu W)\,\right]_{W_0}^{0} = \left[\frac{1}{\mu} ln\,(f - r_T - r_s - \mu W)\,\right]_0^{W_0}.$$

We can now define T_e, the extra capacity, as $T_e = r_T + r_S - f$. This is because we claim that under normal conditions, $f = r_T^0 + r_S^0$, such that the waiting list never grows above zero, and that the additional patients are already on the waiting list. The equation for T_e follows the observation that the current rates of TAVI and SAVR treatment are the normal rates plus the additional capacity.

This substitution allows us to write

$$t_c = \frac{1}{\mu}(ln\,(-T_e - \mu W_0)\ - ln\,(-T_e)\,) = ln\left(1 + \frac{\mu W_0}{T_e}\right)\mu^{-1}.$$

This is the solution we use for calculating the time when the waiting list becomes zero.

We now rely on the assumption that T_e is constant to write

$$m(t_c) = W_0 - T_e t_c.$$

That is, by the time the waiting list is zero, everyone who has not been treated is unfortunately dead.

The assumption of a front-loaded waiting list (i.e., that all additional patients are identified and waiting) is not a strict requirement for this model to be valid. If it is the case that the additional patients are still being identified when the extra capacity is created, then as long as

For peer review only

they are identified at a faster rate than they are treated, the predictions in this model hold. It is only in cases where the identification rate is less than the treatment rate that this assumption becomes invalid. In such cases, T_e can be said to be equal to the identification rate instead. This is true because mortality is not tied to being on the waiting list but from the onset of symptoms. In this way, the waiting list in our model can be thought of as the list of all people who need treatment, even if the NHS is unaware of them.

This model can be extended to predict mortality and time to clear a waiting list for non-constant T_e, but we do not expand on that here.

Supplement 2: Data

We calculate the increase in capacity due to conversions and operational changes as follows. Assume that we increase operations by 20% due to operational changes and convert 10% of all SAVR to TAVI. Also assume that for every three SAVR patients five TAVI patients can be processed. If we convert 10% of SAVR cases to TAVI (783 SAVR patients), we can treat an additional 522 patients from the waiting list. From the 20% increase, we get extra 1039 TAVI and 1566 SAVR operations per year. If we apply 10% conversion to this extra capacity, 156 SAVR operations can be converted into 260 TAVI operations. In total, the operational changes and conversion create an extra capacity of 3232 operations with which to service the waiting list each year: 1822 (1,039+522+261) TAVI and 1410 (1,566-156) SAVR operations.

N.B. We make no assumptions about who the extra TAVI procedures treat, for example, if in the above example, the additional 626 TAVI procedures we gain from conversion (522 from converting the normal capacity and 104 from converting the additional capacity) treated only SAVR patients, the conversion rate would actually be $\frac{626+783+156}{626+1566+78} = 15.6\%$. Normally, we would expect that the application of this extra TAVI would be in the same proportion as the ratio of SAVR to TAVI, which would give a real-world conversion rate of 13.5%.

Supplement 3: App

The app can be accessed at https://github.com/Christian-P-Stickels/AS_Waitinglist_data

For peer review only

Supplement 4: Additional Results

Supplementary figure S1: Heat map of a three-to-four SAVR-to-TAVI conversion

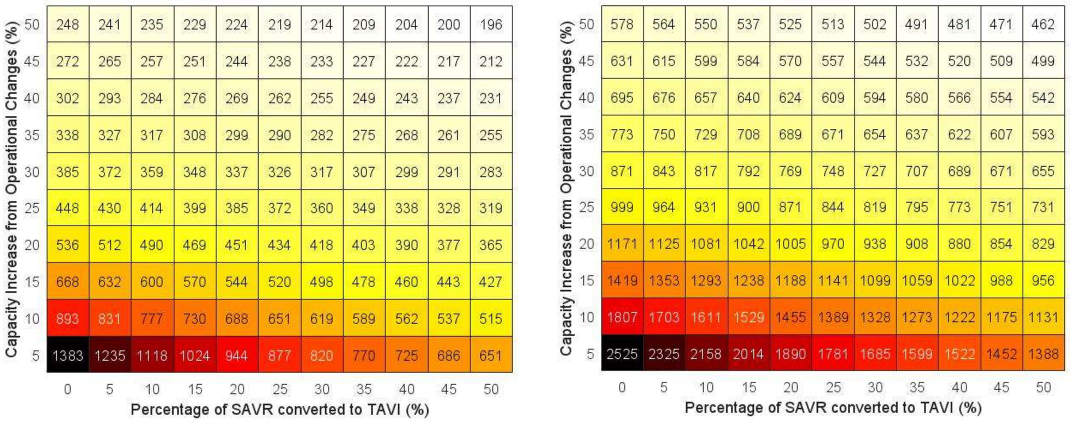

Supplementary Figure S1: Mean time to clear backlog (left) and the resulting deaths (right) as a function of daily percentage increase in capacity (y-axis) and percentage of SAVR converted to TAVI (x-axis), assuming that for every three SAVR operations, four TAVI procedures can be performed instead.

Supplementary figure S2: Heat map of a three-to-five SAVR-to-TAVI conversion

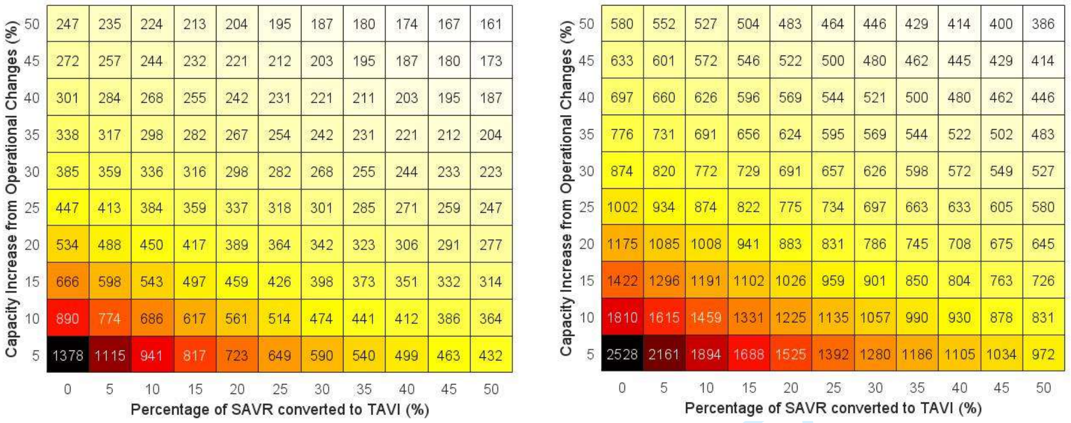

Supplementary Figure S2: Mean time to clear backlog (left) and the resulting deaths (right) as a function of daily percentage increase in capacity (y-axis) and percentage of SAVR converted to TAVI (x-axis), assuming that for every three SAVR operations, five TAVI procedures can be performed instead.

Supplementary figure S3: Heat map of a two-to-four SAVR-to-TAVI conversion

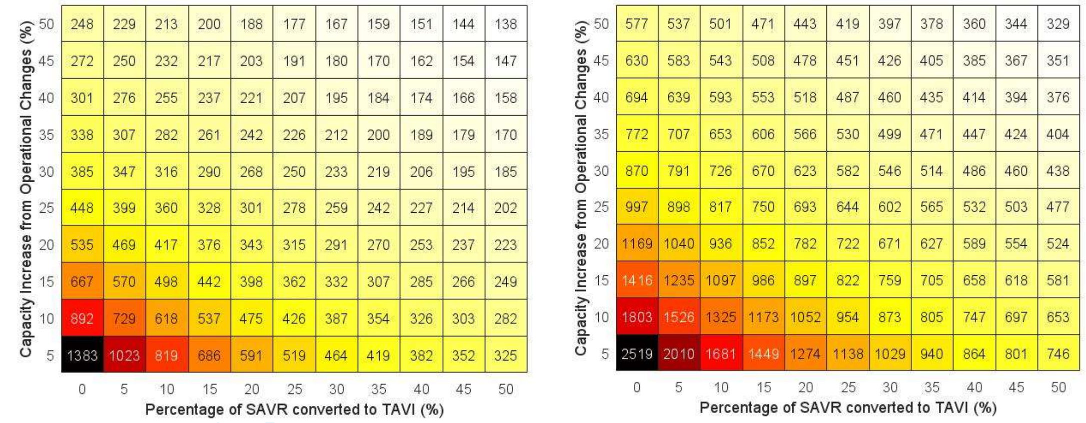

Supplementary Figure S3: Mean time to clear backlog (left) and the resulting deaths (right) as a function of daily percentage increase in capacity (y-axis) and percentage of SAVR converted to TAVI (x-axis), assuming that for every two SAVR operations, four TAVI procedures can be performed instead.

Supplementary figure S4: Error from mortality estimates

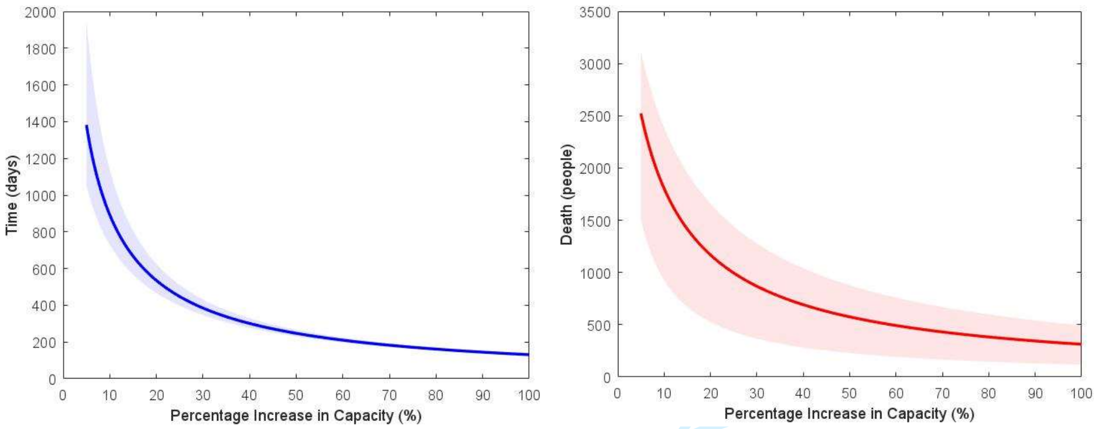

Supplementary figure S4: Time to clear backlog (left) and the resulting deaths (right) with associated 95% confidence intervals as a function of daily percentage increase in capacity, with uncertainty from mortality only. The x-axis is truncated at 5% for visualisation and clarity.

We find that error in the one-year mortality causes higher uncertainty at lower capacity increases, but at higher capacity increases, this uncertainty decreases until it is almost zero with regards to clearance time. This is likely because at higher capacity increases, more of our waiting list clearance comes from treatment, as opposed to death, resulting in less error.

Supplementary figure S5: Error from wait list (W_0) estimates

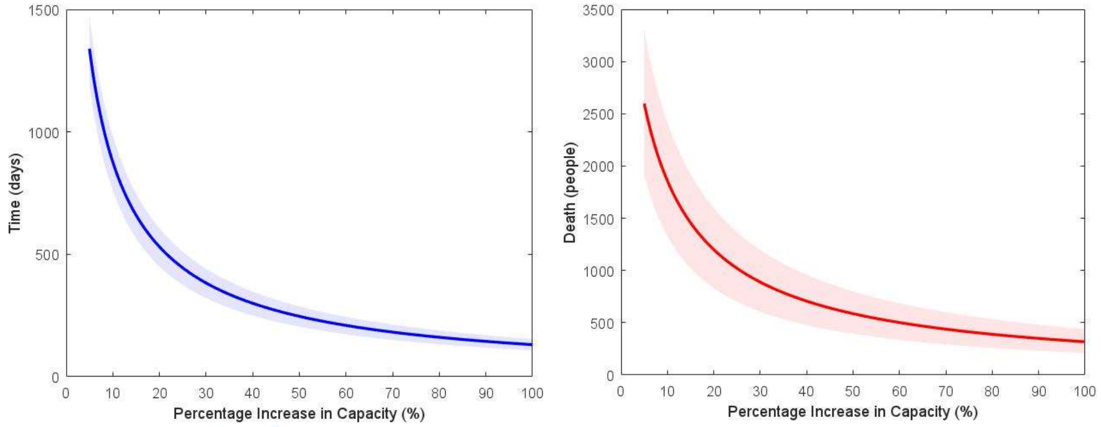

Supplementary figure S5: Time to clear backlog (left) and the resulting deaths (right) with associated 95% confidence intervals as a function of daily percentage increase in capacity, with uncertainty from initial waiting list estimates only. The x-axis is truncated at 5% for visualisation and clarity.

We find that error in the estimate of the wait list length W_0 causes uncertainty that is fairly constant in the time it takes to clear the backlog and in resultant deaths. This is to be expected as we can show that the uncertainty scales with $\ln W_0$. There is a small decrease in uncertainty as we increase capacity, once again because an increase in capacity results in more control of the waiting list reduction.

For peer review only

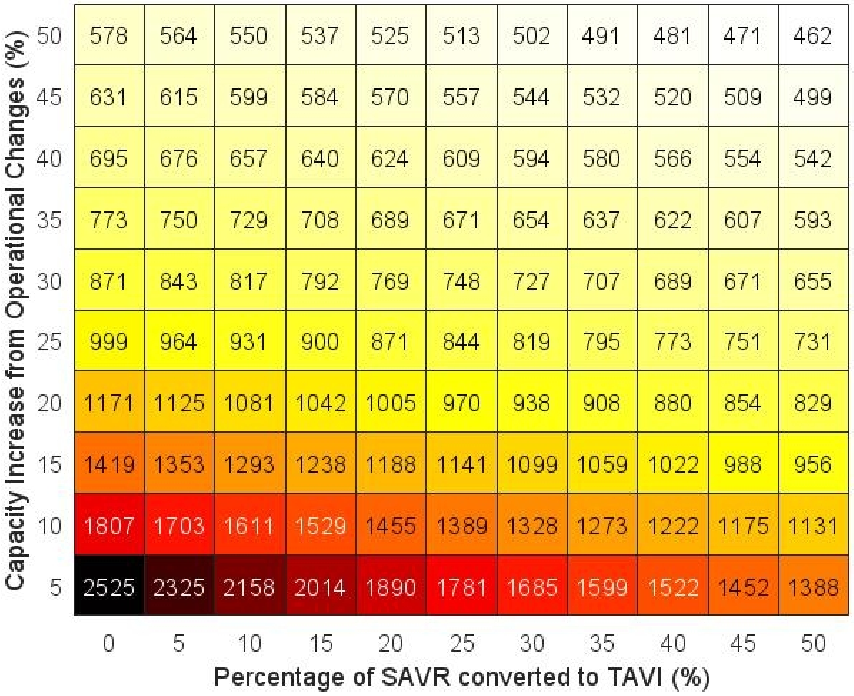

246x185mm (72 x 72 DPI)

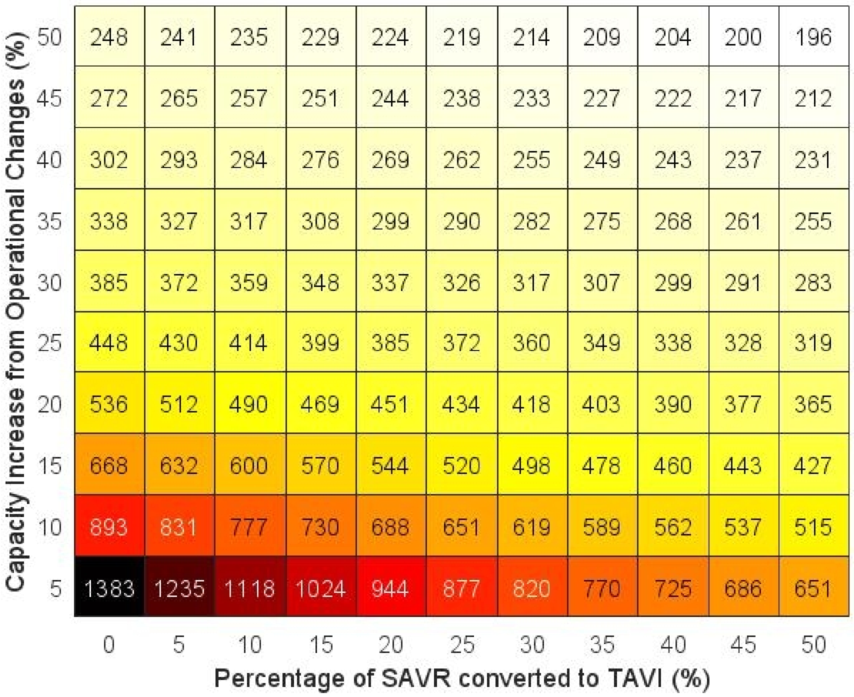

246x185mm (72 x 72 DPI)

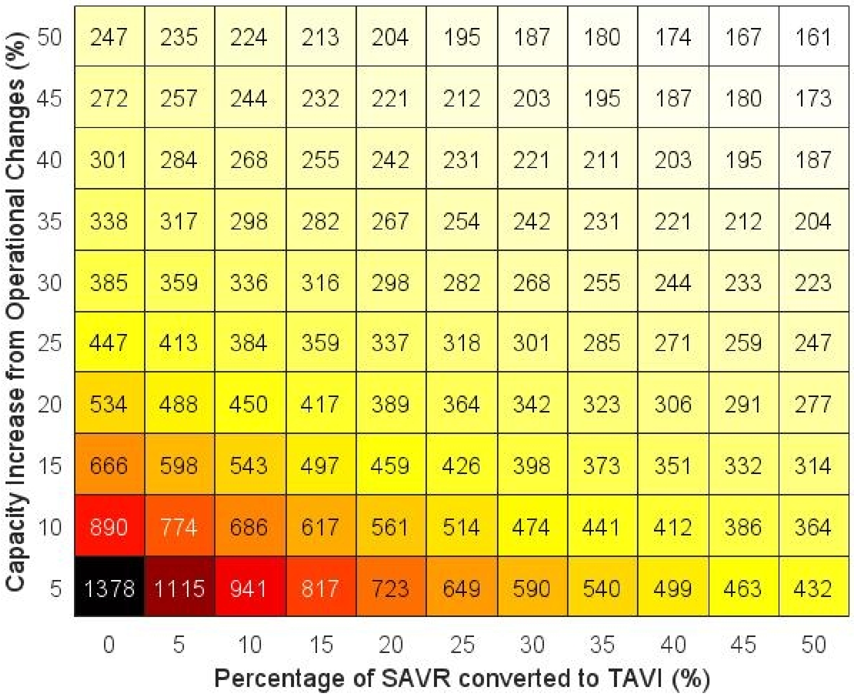

246x185mm (72 x 72 DPI)

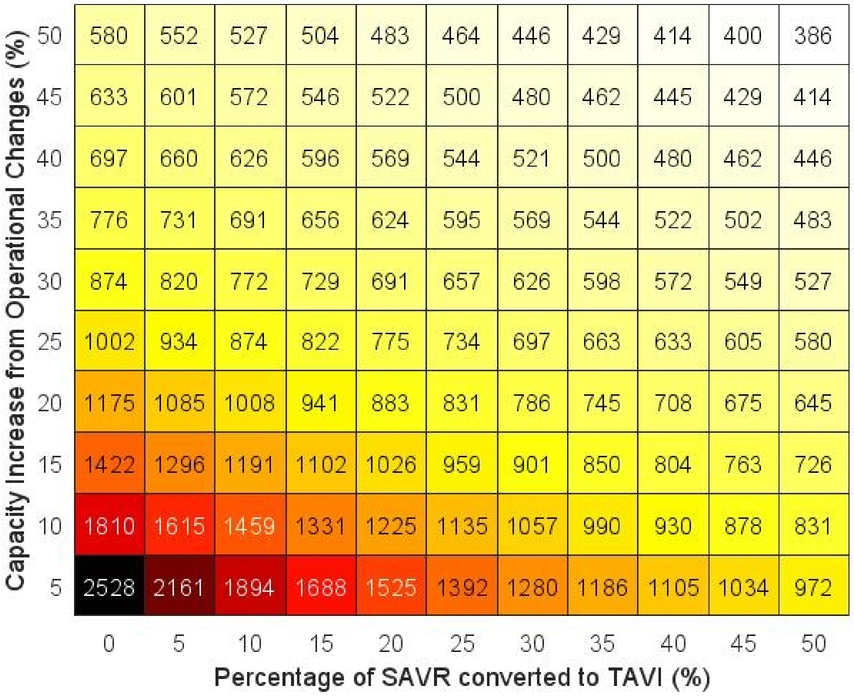

246x185mm (72 x 72 DPI)

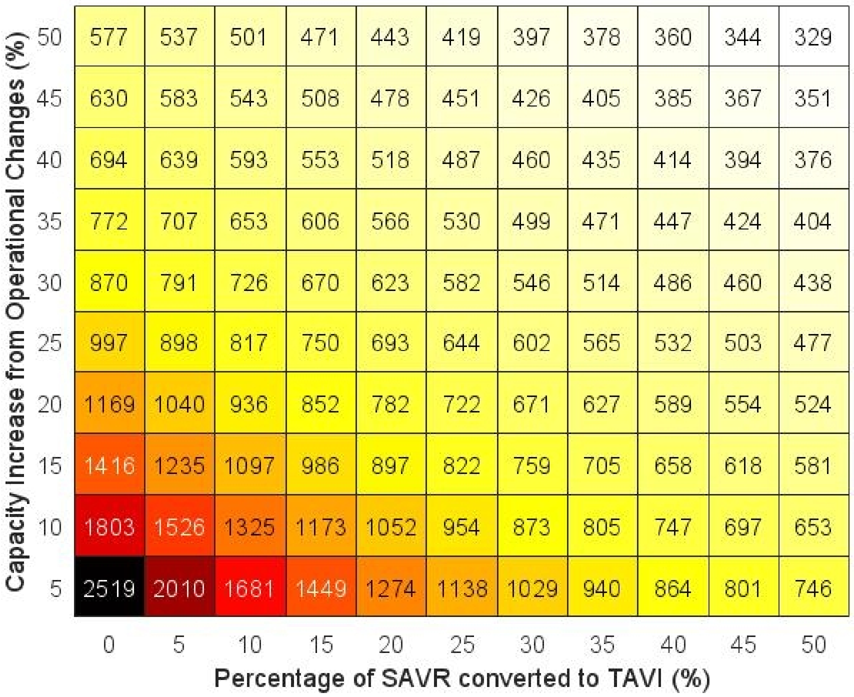

246x185mm (72 x 72 DPI)

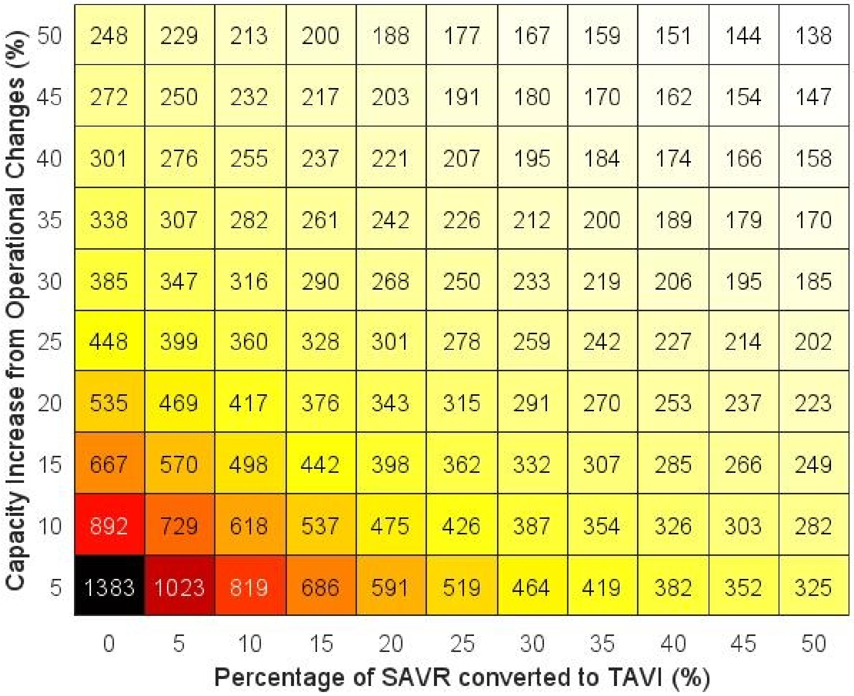

246x185mm (72 x 72 DPI)

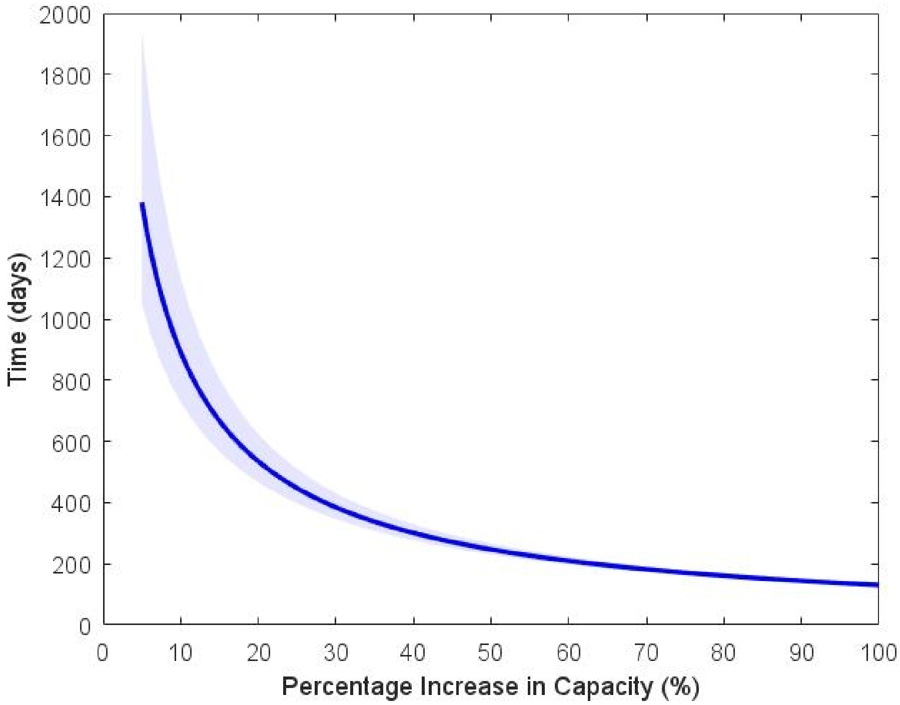

246x185mm (72 x 72 DPI)

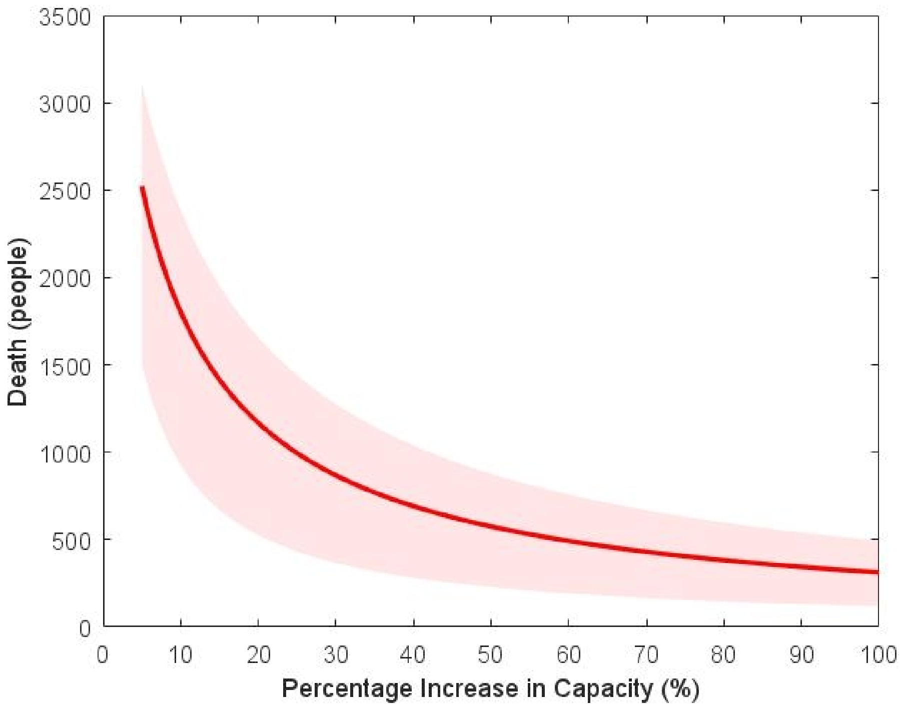

246x185mm (72 x 72 DPI)

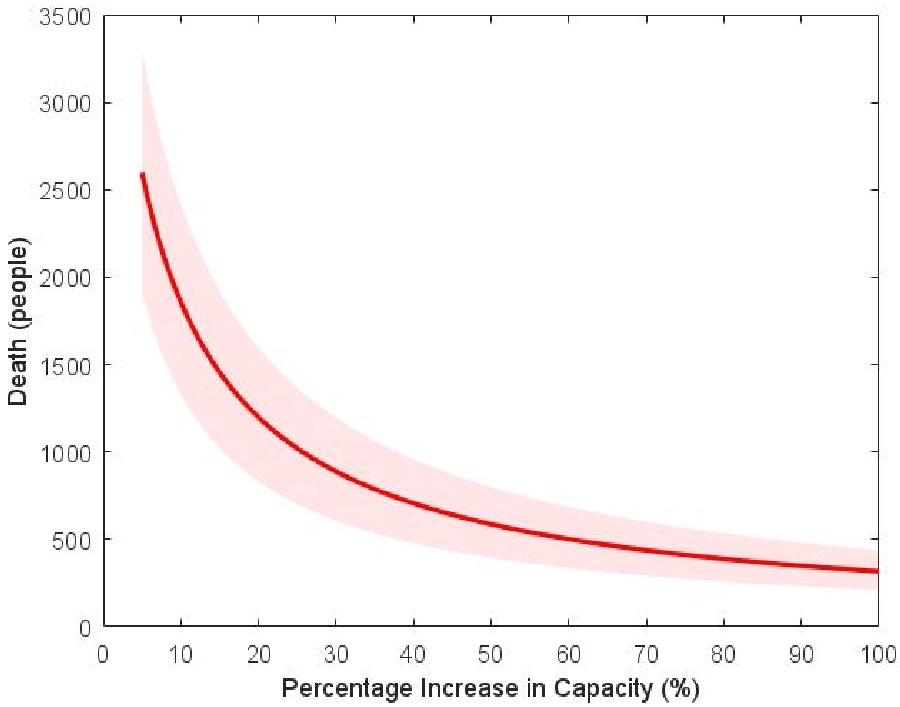

246x185mm (72 x 72 DPI)

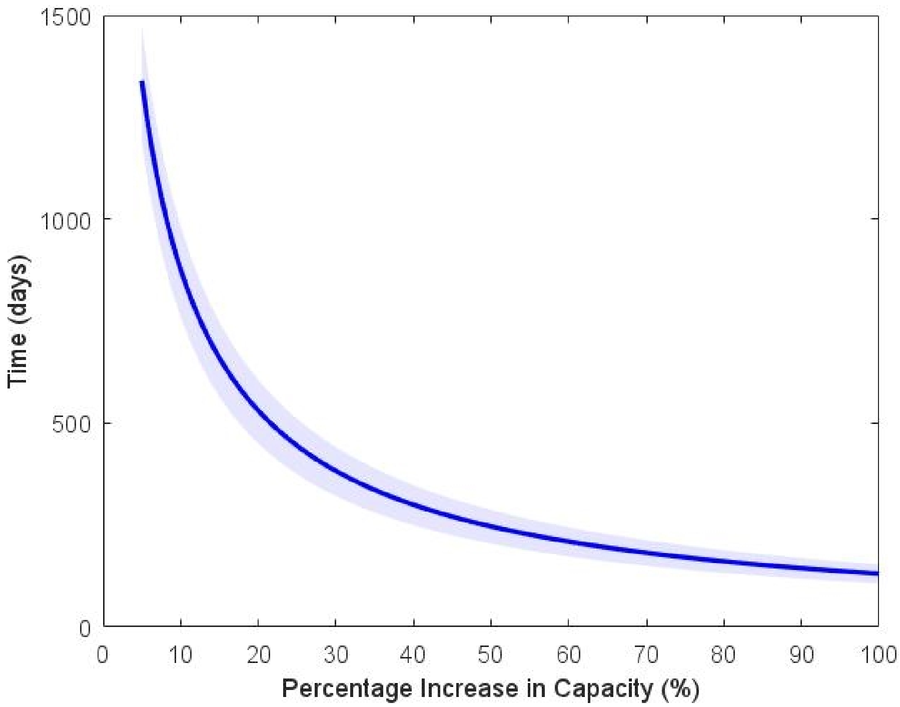

246x185mm (72 x 72 DPI)

BMJ Open

Aortic stenosis post-COVID-19: A mathematical model on waiting lists and mortality

Journal:	*BMJ Open*
Manuscript ID	bmjopen-2021-059309.R1
Article Type:	Original research
Date Submitted by the Author:	09-May-2022
Complete List of Authors:	Stickels, Christian; University of Liverpool, Department of Mathematics<br>Nadarajah, Ramesh; University of Leeds, Leeds Institute for Data Analytics; University of Leeds, Leeds Institute of Cardiovascular and Metabolic Medicine<br>Gale, Chris; University of Leeds, Leeds Institute for Data Analytics; University of Leeds, Leeds Institute of Cardiovascular and Metabolic Medicine<br>Jiang, Houyuan; University of Cambridge, Judge Buisness School<br>Sharkey, Kieran J; University of Liverpool, Department of Mathematical Sciences<br>Gibbison, Ben; Bristol Medical School, Cardiac Anaesthesia and Intensive Care<br>Holliman, Nick; Newcastle University, School of Computing<br>Lombardo, Sara; Loughborough University, Mathematical Sciences<br>Schewe, Lars; The University of Edinburgh School of Mathematics<br>Sommacal, Matteo; Northumbria University, Department of Mathematics, Physics and Electrical Engineering<br>Sun, Louise; University of Ottawa Heart Institute, Division of Cardiac Anesthesiology; Institute for Clinical Evaluative Sciences,<br>Weir-McCall, Jonathan; University of Cambridge, Department of Radiology<br>Cheema, Katherine; British Heart Foundation<br>Rudd, James H F; University of Cambridge, Department of Radiology<br>Mamas, Mamas; Keele University, Keele Cardiovascular Research Group<br>Erhun, Feryal; University of Cambridge
**Primary Subject Heading**:	Cardiovascular medicine
Secondary Subject Heading:	Medical management, Public health
Keywords:	Cardiology < INTERNAL MEDICINE, COVID-19, Valvular heart disease < CARDIOLOGY

SCHOLARONE™
Manuscripts

I, the Submitting Author has the right to grant and does grant on behalf of all authors of the Work (as defined in the below author licence), an exclusive licence and/or a non-exclusive licence for contributions from authors who are: i) UK Crown employees; ii) where BMJ has agreed a CC-BY licence shall apply, and/or iii) in accordance with the terms applicable for US Federal Government officers or employees acting as part of their official duties; on a worldwide, perpetual, irrevocable, royalty-free basis to BMJ Publishing Group Ltd ("BMJ") its licensees and where the relevant Journal is co-owned by BMJ to the co-owners of the Journal, to publish the Work in this journal and any other BMJ products and to exploit all rights, as set out in our licence.

The Submitting Author accepts and understands that any supply made under these terms is made by BMJ to the Submitting Author unless you are acting as an employee on behalf of your employer or a postgraduate student of an affiliated institution which is paying any applicable article publishing charge ("APC") for Open Access articles. Where the Submitting Author wishes to make the Work available on an Open Access basis (and intends to pay the relevant APC), the terms of reuse of such Open Access shall be governed by a Creative Commons licence – details of these licences and which Creative Commons licence will apply to this Work are set out in our licence referred to above.

Other than as permitted in any relevant BMJ Author's Self Archiving Policies, I confirm this Work has not been accepted for publication elsewhere, is not being considered for publication elsewhere and does not duplicate material already published. I confirm all authors consent to publication of this Work and authorise the granting of this licence.

For peer review only

Title

Aortic stenosis post-COVID-19: A mathematical model on waiting lists and mortality

Publication Type

Original Research

Authors

Christian P Stickels[1], Ramesh Nadarajah[2,3,4], Chris P Gale[2,3,4], Houyuan Jiang[5], Kieran J Sharkey[1], Ben Gibbison[6], Nick Holliman[7], Sara Lombardo[8], Lars Schewe[9], Matteo Sommacal[10], Louise Sun[11,12,13], Jonathan Weir-McCall[14], Katherine Cheema[15], James H F Rudd[16], Mamas A. Mamas[17], Feryal Erhun[5]

[1] Department of Mathematical Sciences, University of Liverpool, UK

[2] Leeds Institute for Cardiovascular and Metabolic Medicine, University of Leeds, UK

[3] Leeds Institute of Data Analytics, University of Leeds, UK

[4] Department of Cardiology, Leeds Teaching Hospitals NHS Trust, Leeds, UK

[5] Judge Business School, University of Cambridge, UK

[6] Cardiac Anaesthesia and Intensive Care, Bristol Medical School, University of Bristol, UK

[7] School of Computing, Newcastle University, UK

[8] Mathematical Sciences, Loughborough University, UK

[9] School of Mathematics, University of Edinburgh, UK

[10] Department of Mathematics, Physics and Electrical Engineering, Northumbria University, UK

[11] Division of Cardiac Anaesthesiology, University of Ottawa Heart Institute, Canada

[12] School of Epidemiology and Public Health, University of Ottawa, Canada

[13] Institute for Clinical Evaluative Sciences, Canada

[14] Department of Radiology, University of Cambridge, UK

[15] British Heart Foundation, UK

[16] Division of Cardiovascular Medicine, University of Cambridge, UK

[17] Keele Cardiovascular Research Group, Keele University, UK

Correspondence

Feryal Erhun

Judge Business School

For peer review only - http://bmjopen.bmj.com/site/about/guidelines.xhtml

University of Cambridge
Cambridge, UK
Email: f.erhun@jbs.cam.ac.uk

Word Count
2980

Keywords
Cardiology
COVID-19
Valvular heart disease

For peer review only

For peer review only - http://bmjopen.bmj.com/site/about/guidelines.xhtml

Abstract

Objectives

To provide estimates for how different treatment pathways for the management of severe aortic stenosis (AS) may affect NHS England waiting list duration and associated mortality.

Design

We constructed a mathematical model of the excess waiting list and found the closed-form analytic solution to that model. From published data, we calculated estimates for how the following strategies may affect the time to clear the backlog of patients waiting for treatment and the associated waiting list mortality.

Setting

The NHS in England.

Participants

Estimated aortic stenosis patients in England.

Interventions

1) Increasing the capacity for the treatment of severe AS, 2) converting proportions of cases from surgery to transcatheter aortic valve implantation, and 3) a combination of these two.

Results

In a capacitated system, clearing the backlog by returning to pre-COVID-19 capacity is not possible. A conversion rate of 50% would clear the backlog within 666 (533–848) days with 1419 (597–2189) deaths whilst waiting during this time. A 20% capacity increase would require 535 (434–666) days, with an associated mortality of 1172 (466–1859). A combination of converting 40% cases and increasing capacity by 20% would clear the backlog within a year (343 (281–410) days) with 784 (292–1324) deaths whilst awaiting treatment.

For peer review only - http://bmjopen.bmj.com/site/about/guidelines.xhtml

Conclusion

A strategy change to the management of severe AS is required to reduce the NHS backlog and waiting list deaths during the post-COVID-19 ‘recovery’ period. However, plausible adaptations will still incur a substantial wait to treatment and many hundreds dying whilst waiting.

For peer review only

For peer review only - http://bmjopen.bmj.com/site/about/guidelines.xhtml

Strengths and limitations of this study

- Our model provides a good basis from which to alleviate a time-critical health system problem when data gathering is likely to result in a greater number of deaths.
- Offering TAVI to some SAVR patients in what might be considered sub-optimal per-patient treatment in ideal conditions, could result in better target population-based outcomes.
- The assumption that the entire NHS can be modelled as a single entity with a single waiting list is a limitation of this study.
- We recognise that the waiting numbers used in our study are only estimates because we do not know how many patients with AS died due to COVID-19 infection.

For peer review only

For peer review only - http://bmjopen.bmj.com/site/about/guidelines.xhtml

Introduction

The COVID-19 pandemic has led to the reorganisation of healthcare services to limit the transmission of the virus and deal with the sequelae of infection. This reorganisation had a detrimental effect on cardiovascular services, with reductions in hospitalisations for acute cardiovascular events and the deferral of all but the most urgent interventional procedures and operations.[1, 2]

Aortic stenosis (AS) is the most common form of valvular heart disease. Once stenosis is severe, symptoms follow and the prognosis is poor, with 50% mortality within two years of symptom onset.[3] Thus, timely treatment is of paramount importance. Surgical aortic valve replacement (SAVR) has historically been the default treatment strategy. However, transcatheter aortic valve implantation (TAVI) has recently emerged as an effective and increasingly utilised option across operative risk strata.[4-8]

There was a large decline in TAVI and SAVR procedural activity to treat severe AS during the COVID-19 pandemic.[9] Between the period March to November 2020, it is estimated that the decrease in activity accounted for 4989 (95% CI. 4020–5959) patients in England with severe AS left untreated by TAVI or SAVR.[9] As we move into an era of ‘living with’ COVID-19, plans must urgently be put in place to best manage the additional waiting list burden for treatment of severe AS.[10]

In this study, we used mathematical methods to examine the extent to which additional capacity to provide treatment of severe AS should be created to clear the backlog and minimise deaths of people on the waiting list.

Methods

Study population and assumptions

Data from the UK TAVR registry and NICOR (National Institute for Cardiovascular Outcomes Research) National Adult Cardiac Surgery Audit (NACSA) between 2017 and 2020 have previously been extracted to estimate an excess waiting list size (W_0) of 4989

For peer review only - http://bmjopen.bmj.com/site/about/guidelines.xhtml

(95% CI, 4020–5959) patients with severe AS left untreated as of November 2020.[9] In the absence of contemporaneous data on waiting lists and SAVR and TAVI activity, we have taken this number as the excess backlog on which to model solutions. The incidence of AS has not increased over recent years.[11] Therefore, we assumed that the system was in a steady state before the COVID-19 pandemic and without loss of generality defined the steady-state waiting list to be zero. Additionally, we assumed that the normal rate of flow (f) of new patients into the waiting list for treatment of severe AS would be maintained upon the commencement of additional operations. Thus, the extra capacity that we model is to clear the excess post-COVID-19 backlog.

We took one-year mortality (μ) after the onset of symptoms in severe AS to be 36% (95% CI, 12% – 60%).[12] More recent studies have estimated the one-year mortality to be 51%[5] and 55%, but these included cohorts that were considered inappropriate for SAVR, thus, we considered these estimates unrepresentative of an unselected population with severe AS.[13] The routine capacity for treatment of severe AS was taken from the pre-pandemic period. In 2018/19, the NHS in England performed 7830 SAVR ($r_S^0 = 7830$) and 5197 TAVI ($r_T^0 = 5197$) procedures, for a total throughput of about 13,000 per year.[14]

Modelling

Patients on the waiting list for treatment of severe AS were represented as a dynamical system (figure 1).

To this model, we introduced capacity in surplus to the 2018/19 performance and called this capacity T_e (further details are provided in supplementary material). We assumed that the typical caseload for which the NHS in England can deal with continues; i.e., we assumed that the system will return to pre-pandemic levels first using its baseline capabilities. The backlog accumulated during the pandemic is only reduced by treating patients with this extra capacity or by patient mortality before receiving treatment. We also considered patients in the backlog and patients new to the waiting list indistinguishable. Accordingly, the waiting list size represents the excess number of people seeking treatment who are unable to be treated immediately at any one time. We also assumed that other paths out of the waiting list (i.e. patients seeking private treatment) would be so small in comparison to the uncertainty estimates as to be negligible on the results of our analysis.

For peer review only - http://bmjopen.bmj.com/site/about/guidelines.xhtml

These assumptions were brought together to give an estimated time (see supplementary material for derivation) to clear the waiting list (t_C)

$$t_C = \frac{ln\left(1 + \frac{W_0 \mu}{T_e}\right)}{\mu} \quad (1)$$

and associated mortality ($m(t_C)$)

$$m(t_C) = W_0 - T_e t_C. \quad (2)$$

Using equations (1) and (2), we predicted the length of time and associated mortality for different percentage increases in capacity. We assumed any capacity increase to be constant throughout the entire modelled period. For example, if we increased daily capacity by 5% this would result in, $T_e = \frac{r_S^0 + r_T^0}{365} * 5\% = 1.785$ extra procedures per day, across the whole of the NHS in England. We generated 10,000 random values for the one-year mortality rate and initial waiting list length. We assumed that the uncertainty in both variables was normally distributed. For every T_e, we present the mean and the 2.5 and 97.5 percentiles of the 10,000 simulations for time to clear the waiting list and the associated mortality. That is, we present the 95% reference range.[15]

Interventions and outcomes

We investigated three types of capacity increase: 1) a general increase in the capacity to provide SAVR and TAVI, which could be facilitated by an increased number of procedures per list, additional lists, and prioritisation of care pathways and staffing to treat severe AS; 2) extra capacity created by treating some patients with TAVI who would routinely have SAVR; 3) a combination of a general increase in capacity and the conversion of a proportion of cases from SAVR to TAVI. During the COVID-19 pandemic, TAVI was performed in patients usually referred for surgery, with no difference in short term outcomes compared to historical reference groups.[16, 17]

We assumed that the duration of a SAVR would routinely be between 2-4 hours and a TAVI between 1-2 hours.[18, 19] As such, we assumed within the time for two SAVR operations, three TAVI could be performed instead.[20] Several clinical factors may favour SAVR over TAVI (including concomitant severe coronary artery disease, low STS score, bicuspid aortic

For peer review only - http://bmjopen.bmj.com/site/about/guidelines.xhtml

For peer review only

valve etc.); therefore, we assumed that, in the short term, no more than 50% of patients could be converted from SAVR to TAVI.[21] We also assumed that no more than 50% extra capacity could be created by other means (e.g. extra lists, more procedures per list). We simulated two principal outcomes based on the creation of additional capacity (T_e): the time to clear the backlog (reduce to zero), and the mortality of patients within the excess backlog whilst on the waiting list to be treated.

We completed additional sensitivity analyses for how the conversion of SAVR to TAVI could affect the principal outcomes, including if three SAVR operations could be routinely completed in a day and four to five TAVI procedures per day (presuming increasing uptake of a minimalist TAVI approach without general anaesthesia enabling more rapid procedure time).[22]

Patient and public involvement

Patients and the public were not involved in the conduct of this study.

Results

In the pre-COVID-19 period, the routine capacity for treatment of severe AS was set to cover the normal incident rate. That is, clearing the backlog by returning to pre-COVID-19 capacity is not possible. As a result, mortality on the excess waiting list at one year are estimated to be more than 1500, putting a strong emphasis on the need for change.

Total additional capacity

Figure 2 provides simulations of the time to clear the excess backlog and the mortality of patients on the waiting list based on the amount of total additional capacity, T_e. With a 5% increase in the capacity to provide treatment of severe AS, we estimate it would take 1384 (1025–1994) days to clear the excess backlog, with 2526 (1355–3516) deaths. A 20% increase in total capacity would provide a benefit in clearing the excess backlog within 536 (434–666) days, with an estimate of 1173 (466–1859) deaths. As total capacity increased further, there was a diminishing return in clearing the backlog and avoiding associated mortality; the greater the capacity increase, the fewer lives are saved for every extra increase

For peer review only - http://bmjopen.bmj.com/site/about/guidelines.xhtml

in capacity. Even if it was possible to double capacity, it was estimated that it may take 131 (126–137) days to clear the backlog and there would be 313 (118–494) deaths on the waiting list.

The effect of converting SAVR to TAVI

The conversion of a proportion of cases from surgery to TAVI provides a modest improvement in estimates of time to clear the backlog and mortality on the waiting list. With the conversion of 30% of SAVR operations to TAVI procedures, without the creation of additional capacity in the system, we estimated that it would take 975 (741–1284) days to clear the backlog and there would be 1914 (923–2809) deaths on the waiting list. Even with a conversion of 50% of SAVR operations to TAVI procedures, the estimated backlog would be cleared within 666 (533–848) days with 1419 (597–2189) deaths. For the highest conversion ratio that we considered (2:4), at a 50% rate of conversion, we estimated the backlog to be cleared in 384 (330–462) days with 871 (314–1426) deaths. Whilst this result is improved, we consider a 2:4 conversion ratio the highest reasonable ratio in the short-term, and is unlikely to be achieved at every centre immediately. It is also worth noting that even if this was achieved, the backlog would still take over a year to clear.

Combining conversion of SAVR to TAVI and additional capacity

Figures 3a and 3b demonstrate the range of possibilities in creating extra capacity. Each line demonstrates a range of intervention strategies that provide the same result. For example, to reduce the mean predicted deaths to 1000 (red line figure 3b), centres could increase capacity to provide an extra 25% procedures per week at the same mix as pre-pandemic, or they could convert 50% of SAVR operations to TAVI and increase their capacity by 8.7% at that mix. Figures 3c and 3d represent how the combinations of interventions to increase capacity within the system alongside the conversion of SAVR to TAVI would impact the time to clear the backlog and on the associated mortality of waiting. Mortality on the waiting list is less responsive to our modelled interventions than the time to clear the backlog (the darker coloured regions of figure 3d make up a greater proportion of the estimates than those of figure 3c). Increasing capacity within the system alongside converting a proportion of SAVR cases to TAVI provides the greatest estimated benefit in clearing the backlog and avoiding associated mortality. A combination that would result in the clearance of the backlog within a year might be of interest for decision makers. With the conversion of 40% of SAVR

For peer review only - http://bmjopen.bmj.com/site/about/guidelines.xhtml

operations to TAVI and the creation of an additional 20% capacity, we estimated that the backlog would be cleared in just under a year – 343 days (281–410) with 784 (292–1324) deaths before treatment.

Sensitivity analyses where the number of TAVI procedures that could be completed within the same time as SAVR was altered (TAVI to SAVR: 4 to 3, 4 to 2, 5 to 3) support these findings (supplementary material figures S1 – S3). Furthermore, sensitivity analyses show that with the best-in-class practices (TAVI to SAVR: 4 to 2), even a more modest combination (a conversion of 35% and creation of an additional 10% capacity) may be enough to clear the backlog within a year.

Discussion

In this study, using dynamical system modelling, we provide estimates for how changes to treatment pathways for patients with severe aortic stenosis may affect the time taken to clear the backlog and minimise mortality on the waiting list in the NHS of England. Without providing at least 20% total additional capacity for the interventional treatment of AS, we estimated there would be more than 1000 deaths on the waiting list over a period of nearly 1.5 years. A conversion of cases from SAVR to TAVI would expedite the clearance of the backlog, but even converting half the cases to TAVI would still result in over 1400 deaths over a period of almost 2 years. A combination of converting 40% of cases usually planned for SAVR to TAVI and creating 20% additional capacity for procedures (through measures such as extra lists) would clear the excess backlog within one year, with 784 deaths.

Our study has several strengths. First, in a time-critical clinical situation of many unknowns, our use of novel mathematical models provides plausible estimates on which to base health services planning, and provides an exemplar that may be used in service delivery in other conditions in the post-pandemic landscape. Given the high event rate amongst this population, waiting for more contemporary data to be collected may well not provide enough time to institute system changes to prevent deaths. Second, we also provide specific estimates for how the conversion of cases to TAVI from surgery may affect waiting lists and associated mortality, which can inform local MDT discussions. Third, our model can act as a basis for a

For peer review only - http://bmjopen.bmj.com/site/about/guidelines.xhtml

clinical and cost-benefit analysis to evaluate different ways to increase capacity and define the optimal strategy at each centre. For each centre, the most effective combination of converting SAVR to TAVI and provision or prioritisation of treatment of severe AS can be generated.

We recognise the limitations inherent in modelling a complex situation. First, we represent the NHS in England as a single entity. As such, we implicitly assume that population and capacity are distributed evenly throughout the country by treating centre capacity. If the distribution of waiting list patients deviates significantly from the distribution of treatment centres weighted by capacity, the time it would take to clear the waiting list, and thus the mortality rate would be higher. Second, we have not attempted to calculate how many patients with AS may have died in the COVID-19 pandemic, which could have reduced the numbers of deaths on the waiting list and the duration of the waiting list because of an underestimation of ‘abandonment’ from the model. Third, our assumed mortality rate may differ at a centre-level due to prioritising clinically more vulnerable patients on the waiting list. Fourth, a centre-level analysis could account for the different practices in each treatment centre and identify strategies that work best for each centre. Fifth, our estimates from converting cases from SAVR to TAVI do not include post-procedural factors such as the requirement for intensive care capacity, hospital stay and further procedures because these rely on multiple centre-specific factors. Finally, it has been shown that rapid growth in the demand for TAVI can overwhelm current capacity,[23] which may lead to prolonged wait times and subsequent adverse outcomes while patients are on the waitlist. Therefore, a demand model that captures the changes of demand for TAVI and SAVR would be a helpful future direction of analysis.

A previous study used a mathematical model to quantify the cumulative cardiac surgical backlog (including coronary artery bypass grafting surgery, valve replacement and transcatheter aortic and mitral valve replacements) in two centres based on the projected pandemic duration in the United States of America (USA).[24] The authors used simple mathematical models to predict the time required to clear the backlog depending on increased operating capacity. However, the authors did not consider mortality, which we have as it is of critical importance to patients and when planning services.

For peer review only - http://bmjopen.bmj.com/site/about/guidelines.xhtml

The results of our study highlight concerns pertaining to the deferral of non-emergency treatment for severe AS during the ‘recovery period’ of COVID-19. Severe AS is a progressive condition with valve replacement the only available treatment improving prognosis.[25] On a local, regional, and national scale, healthcare systems will need to examine capacity, set priorities, and plan for adequate capacity to manage the backlog of patients with severe AS. The response will be complicated by prior exhaustion of human resources from the pandemic and competition with other specialities, which will also have backlogs.[26]

Nonetheless, planning should prioritise patients at the highest risk from a deferral of treatment. Mortality on the waiting list for AS has been reported to be as high as 14%.[27] Furthermore, patients awaiting structural procedures deferred due to the pandemic have been found to have significantly higher mortality rates compared to those with stable coronary artery disease.[28] Prioritising capacity for treatment of patients with severe AS may mean reduced capacity for other procedures. Providing 20% extra capacity for TAVI and SAVR may only require the addition of one extra list per week at the expense of other procedures, as many centres only conduct TAVI procedures on between two and three days per week.[22] This interaction will require collaborative decision-making on a local level accepting that these are difficult, imperfect times. We also show that the conversion of a proportion of cases that would usually be managed by SAVR to TAVI can help expedite treatment and reduce mortality on the waiting list. During the pandemic, TAVI procedures were performed in patients usually referred for surgery with no apparent difference in short term outcomes;[16, 17] and data continues to emerge for longer-term efficacy and safety of TAVI across operative risk strata.[29,30] Recent European guidelines suggest that TAVI would be a preferable option for patients over 75 years of age compared to SAVR.[21]

To help planning, we provide an app (https://github.com/Christian-P-Stickels/AS_Waitinglist_data) to explore the impact of alterations in capacity and treatment pathways on waiting lists and mortality related to severe AS at a local, regional and national level (supplementary material).

Conclusions

For peer review only - http://bmjopen.bmj.com/site/about/guidelines.xhtml

In this study, we found that without a combination of increased capacity for treatment of patients with severe aortic stenosis and an expansion in the use of TAVI, there would be many potentially avoidable deaths during the post-COVID-19 recovery period. Our study findings and accompanying app may help inform the planning of cardiac services.

Acknowledgement

We want to thank all the participants of the V-KEMS Study Group on "Modelling Solutions to the Impact of COVID-19 on Cardiovascular Waiting Lists" that took place on February 2-4, 2021, for thought-provoking discussions. Our special thanks to Clare Merritt (Newton Gateway to Mathematics), whose help extended beyond the workshop and was crucial in completing this work, and to Alan Champneys who brought the group together in the first place. BG is supported by the NIHR Bristol Biomedical Research Centre at the University of Bristol and University Hospitals Bristol and Weston NHS Foundation Trust. JHFR is part-supported by the NIHR Cambridge Biomedical Research Centre, the British Heart Foundation, HEFCE, the EPSRC Cambridge Centre for Mathematics of Information in Healthcare and the Wellcome Trust.

Funding

This study was part funded by EPSRC Cambridge Centre for Mathematics of Information in Healthcare, grant number EP/T017961/1. None of the study funding sources had an impact on the design, data analysis, writing of or decision to publish this paper.

Competing Interests

BG acknowledges grants not related to this project from the David Telling Charitable Trust, and the Biotechnology and Biological Sciences Research Council, he additionally declared Associate Editorship of Anesthesia Journal, and being the chair DMSC for the COPIA Trial. All other authors confirm that they have no competing interests to declare.

Data Sharing

For peer review only - http://bmjopen.bmj.com/site/about/guidelines.xhtml

No additional data available

Contributorship statement

MAM proposed the initial workshop and designed the research question, MAM, CPG, RN, BG and JHFR all helped to run said workshop as clinical experts. All members but KC and FE were involved in conceptualisation in the initial workshop. CS, HJ, KS, and FE designed the model with clinical guidance from MAM, CPG, RN, BG and JHFR. CS performed data analysis. CS, RN and FE drafted the initial manuscript. MAM, CPG, BG, JHFR, NH, SL, LaSc, MS, LoSu, JWM, KC provided critical interpretation and revision of the manuscript. All authors approved the final manuscript.

Ethics Statement

This paper did not require ethics approval.

References

[1] Mohamed MO, Banerjee A, Clarke S, *et al.* Impact of COVID-19 on cardiac procedure activity in England and associated 30-day mortality. *European Heart Journal - Quality of Care and Clinical Outcomes* 2021;**7**:247–56. doi:10.1093/ehjqcco/qcaa079

[2] Bhatt AS, Moscone A, McElrath EE, *et al.* Fewer Hospitalizations for Acute Cardiovascular Conditions During the COVID-19 Pandemic. *Journal of the American College of Cardiology* 2020;**76**:280–8. doi:10.1016/j.jacc.2020.05.038

[3] Généreux P, Stone GW, O'Gara PT, *et al.* Natural History, Diagnostic Approaches, and Therapeutic Strategies for Patients with Asymptomatic Severe Aortic Stenosis. *Journal of the American College of Cardiology* 2016;**67**:2263–88. doi:10.1016/j.jacc.2016.02.057

[4] Leon MB, Smith CR, Mack M, *et al.* Transcatheter Aortic-Valve Implantation for Aortic Stenosis in Patients Who Cannot Undergo Surgery. *New England Journal of Medicine* 2010;**363**:1597–607. doi:10.1056/NEJMoa1008232

For peer review only - http://bmjopen.bmj.com/site/about/guidelines.xhtml

[5] Leon MB, Smith CR, Mack MJ, *et al.* Transcatheter or Surgical Aortic-Valve Replacement in Intermediate-Risk Patients. *New England Journal of Medicine* 2016;**374**:1609–20. doi:10.1056/NEJMoa1514616

[6] Smith CR, Svensson LG, Makkar RR, *et al.* Transcatheter versus Surgical Aortic-Valve Replacement in High-Risk Patients. *The New England Journal of Medicine* 2011;:12. doi:10.1056/NEJMoa1103510

[7] Mack MJ, Leon MB, Thourani VH, *et al.* Transcatheter Aortic-Valve Replacement with a Balloon-Expandable Valve in Low-Risk Patients. *New England Journal of Medicine* 2019;**380**:1695–705. doi:10.1056/NEJMoa1814052

[8] Popma JJ, Deeb GM, Yakubov SJ, *et al.* Transcatheter Aortic-Valve Replacement with a Self-Expanding Valve in Low-Risk Patients. *New England Journal of Medicine* 2019;**380**:1706–15. doi:10.1056/NEJMoa1816885

[9] Martin GP, Curzen N, Goodwin AT, *et al.* Indirect Impact of the COVID-19 Pandemic on Activity and Outcomes of Transcatheter and Surgical Treatment of Aortic Stenosis in England. *Circ: Cardiovascular Interventions* 2021;**14**. doi:10.1161/CIRCINTERVENTIONS.120.010413

[10] Ro R, Khera S, Tang GHL, *et al.* Characteristics and Outcomes of Patients Deferred for Transcatheter Aortic Valve Replacement Because of COVID-19. *JAMA Netw Open* 2020;**3**:e2019801. doi:10.1001/jamanetworkopen.2020.19801

[11] Martinsson A, Li X, Andersson C, *et al.* Temporal Trends in the Incidence and Prognosis of Aortic Stenosis: A Nationwide Study of the Swedish Population. *Circulation* 2015;**131**:988–94. doi:10.1161/CIRCULATIONAHA.114.012906

[12] Schwarz F, Baumann P, Manthey J, *et al.* The effect of aortic valve replacement on survival. *Circulation* 1982;**66**:1105–10. doi:10.1161/01.CIR.66.5.1105

For peer review only

For peer review only - http://bmjopen.bmj.com/site/about/guidelines.xhtml

[13] Wenaweser P, Pilgrim T, Kadner A, *et al.* Clinical Outcomes of Patients With Severe Aortic Stenosis at Increased Surgical Risk According to Treatment Modality. *Journal of the American College of Cardiology* 2011;**58**:2151–62. doi:10.1016/j.jacc.2011.05.063

[14] NICOR. National Adult Cardiac Surgery Report, 2020 Summary Report. https://www.nicor.org.uk/national-cardiac-audit-programme/adult-cardiac-surgery-surgery-audit/ (Accessed 17 September 2021)

[15] 4. Statements of probability and confidence intervals | The BMJ. https://www.bmj.com/about-bmj/resources-readers/publications/statistics-square-one/4-statements-probability-and-confiden (accessed 24 Mar 2022).

[16] Adlam D, Chan N, Baron J, *et al.* Aortic stenosis in the time of COVID-19: Development and outcomes of a rapid turnaround TAVI service. *Catheter Cardiovasc Interv* 2021;**98**. doi:10.1002/ccd.29550

[17] Shafi AMA, Awad WI. Transcatheter aortic valve implantation versus surgical aortic valve replacement during the COVID-19 pandemic—Current practice and concerns. *J Card Surg* 2021;**36**:260–4. doi:10.1111/jocs.15182

[18] Aortic valve replacement. nhs.uk. 2017. https://www.nhs.uk/conditions/aortic-valve-replacement/ (accessed 13 Oct 2021).

[19] TAVI (Transcatheter aortic valve implantation). https://www.bhf.org.uk/informationsupport/treatments/tavi (accessed 13 Oct 2021).

[20] Martin JA, Mayhew CR, Morris AJ, *et al.* Using Time-Driven Activity-Based Costing as a Key Component of the Value Platform: A Pilot Analysis of Colonoscopy, Aortic Valve Replacement and Carpal Tunnel Release Procedures. *J Clin Med Res* 2018;**10**:314–20. doi:10.14740/jocmr.v10i4.3350

For peer review only

For peer review only - http://bmjopen.bmj.com/site/about/guidelines.xhtml

[21] Vahanian A, Beyersdorf F, Praz F, *et al.* 2021 ESC/EACTS Guidelines for the management of valvular heart disease. *European Heart Journal* 2021;:ehab395. doi:10.1093/eurheartj/ehab395

[22] Ali N, Faour A, Rawlins J, *et al.* 'Valve for Life': tackling the deficit in transcatheter treatment of heart valve disease in the UK. *Open Heart* 2021;**8**:e001547. doi:10.1136/openhrt-2020-001547

[23] Henning KA, Ravindran M, Qiu F, *et al.* Impact of procedural capacity on transcatheter aortic valve replacement wait times and outcomes: a study of regional variation in Ontario, Canada. *Open Heart* 2020;**7**:e001241. doi: 10.1136/openhrt-2020-001241

[24] Salenger R, Etchill EW, Ad N, *et al.* The Surge After the Surge: Cardiac Surgery Post–COVID-19. *The Annals of Thoracic Surgery* 2020;**110**:2020–5. doi:10.1016/j.athoracsur.2020.04.018

[25] Khialani B, MacCarthy P. Transcatheter management of severe aortic stenosis during the COVID-19 pandemic. *Heart* 2020;**106**:1183–90. doi:10.1136/heartjnl-2020-317221

[26] Perdoncin E, Greenbaum AB, Grubb KJ, *et al.* Safety of same-day discharge after uncomplicated, minimalist transcatheter aortic valve replacement in the COVID-19 era. *Catheter Cardiovasc Interv* 2021;**97**:940–7. doi:10.1002/ccd.29453

[27] Nuis RJ, Dager AE, van der Boon RM, *et al.* Patients with aortic stenosis referred for TAVI: treatment decision, in-hospital outcome and determinants of survival. *Neth Heart J* 2012;**20**:16–23. doi:10.1007/s12471-011-0224-z

[28] Moreno R, Díez J, Diarte J, *et al.* Consequences of canceling elective invasive cardiac procedures during Covid-19 outbreak. *Catheter Cardiovasc Interv* 2021;**97**:927–37. doi:10.1002/ccd.29433

For peer review only - http://bmjopen.bmj.com/site/about/guidelines.xhtml

[29] Blackman DJ, Saraf S, MacCarthy PA, *et al.* Long-Term Durability of Transcatheter Aortic Valve Prostheses. *Journal of the American College of Cardiology* 2019;**73**:537–45. doi:10.1016/j.jacc.2018.10.078

[30] Jørgensen TH, Thyregod HGH, Ihlemann N, *et al.* Eight-year outcomes for patients with aortic valve stenosis at low surgical risk randomized to transcatheter vs. surgical aortic valve replacement. *European Heart Journal* 2021;**42**:2912–9. doi:10.1093/eurheartj/ehab375

Legends and Captions

Figure 1: Dynamical system model of the waiting list length.

Figure 2: Time to clear backlog (left) and the resulting deaths (right) with associated 95% confidence intervals as a function of daily percentage increase in capacity, with uncertainty from mortality and the initial waiting list. The x-axis is truncated at 5% for visualisation and clarity.

Figure 3: Mean time to clear backlog (left) and the resulting deaths (right) as a function of daily percentage increase in capacity (y-axis) and percentage of SAVR converted to TAVI (x-axis) (Presented in two different forms). A) Isoclines of constant mean clearance-time going from half a year (blue) to 2 years (purple) in half-year increments. B) Isoclines of constant mean mortality after clearing the backlog from 500 people (blue) to 2000 (purple) in 500-person increments. C) Heatmap of different combinations of conversion and daily capacity increases and how long the backlog would take to clear on average, in days. D) Heatmap of different combinations of conversion and daily capacity increases and how many people would die, on average.

For peer review only - http://bmjopen.bmj.com/site/about/guidelines.xhtml

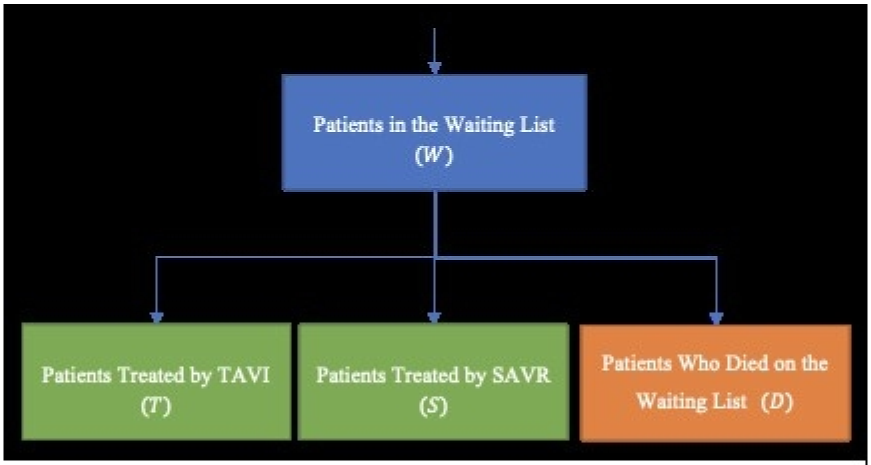

Figure 2 Dynamical system model of the waiting list length.

Figure 1: Dynamical system model of the waiting list length

114x67mm (96 x 96 DPI)

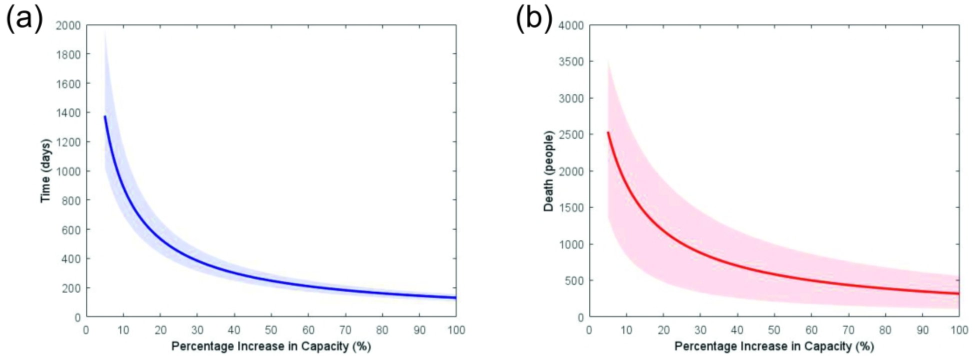

Figure 2: Time to clear backlog (left) and the resulting deaths (right) with associated 95% confidence intervals as a function of daily percentage increase in capacity, with uncertainty from mortality and the initial waiting list. The x-axis is truncated at 5% for visualisation and clarity.

272x97mm (300 x 300 DPI)

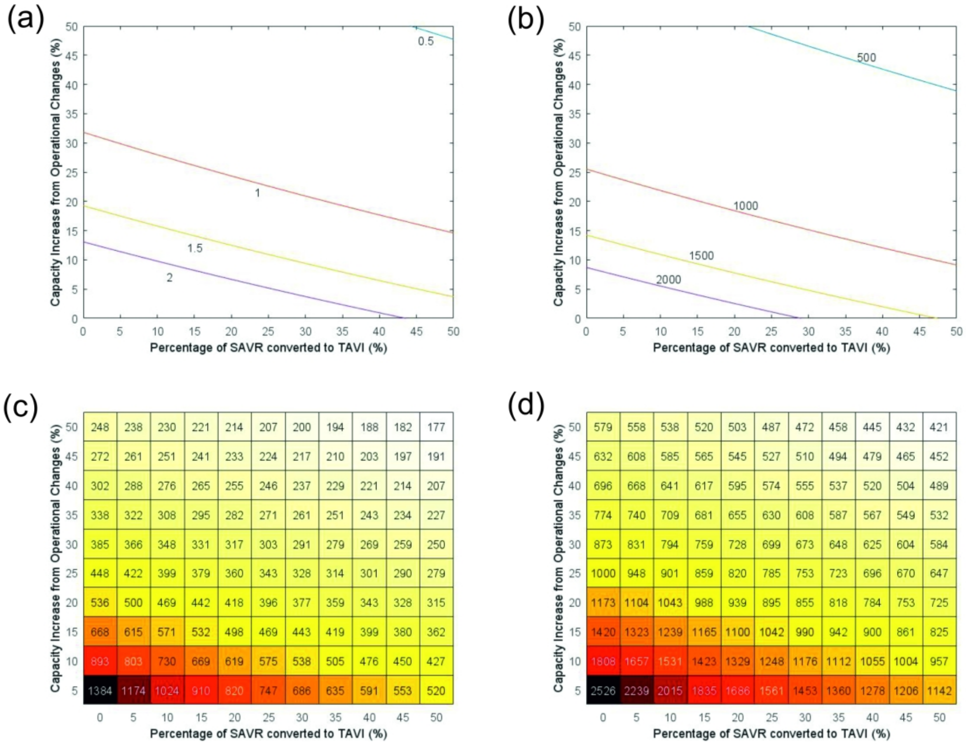

Figure 3: Mean time to clear backlog (left) and the resulting deaths (right) as a function of daily percentage increase in capacity (y-axis) and percentage of SAVR converted to TAVI (x-axis) (Presented in two different forms). A) Isoclines of constant mean clearance-time going from half a year (blue) to 2 years (purple) in half-year increments. B) Isoclines of constant mean mortality after clearing the backlog from 500 people (blue) to 2000 (purple) in 500-person increments. C) Heatmap of different combinations of conversion and daily capacity increases and how long the backlog would take to clear on average, in days. D) Heatmap of different combinations of conversion and daily capacity increases and how many people would die, on average.

272x202mm (300 x 300 DPI)

SUPPLEMENTS

Supplement 1: Mathematical Derivation of the Differential Equation and its Solution

From figure 1, we can write the following equation:

$$\frac{dW}{dt} = f - r_T - r_S - \mu W.$$

We can then re-write and integrate this equation

$$\int_0^{t_c} 1dt = \int_{W_0}^{0} \frac{1}{f - r_T - r_S - \mu W} dW$$

$$t_c = \left[-\frac{1}{\mu} ln\,(f - r_T - r_s - \mu W)\right]_{W_0}^{0} = \left[\frac{1}{\mu} ln\,(f - r_T - r_s - \mu W)\right]_{0}^{W_0}.$$

We can now define T_e, the extra capacity, as $T_e = r_T + r_S - f$. This is because we claim that under normal conditions, $f = r_T^0 + r_S^0$, such that the waiting list never grows above zero, and that the additional patients are already on the waiting list. The equation for T_e follows the observation that the current rates of TAVI and SAVR treatment are the normal rates plus the additional capacity.

This substitution allows us to write

$$t_c = \frac{1}{\mu}(ln\,(-T_e - \mu W_0)\ - ln\,(-T_e)\,) = ln\left(1 + \frac{\mu W_0}{T_e}\right)\mu^{-1}.$$

This is the solution we use for calculating the time when the waiting list becomes zero.

We now rely on the assumption that T_e is constant to write

$$m(t_c) = W_0 - T_e t_c.$$

That is, by the time the waiting list is zero, everyone who has not been treated is unfortunately dead.

The assumption of a front-loaded waiting list (i.e., that all additional patients are identified and waiting) is not a strict requirement for this model to be valid. If it is the case that the additional patients are still being identified when the extra capacity is created, then as long as

they are identified at a faster rate than they are treated, the predictions in this model hold. It is only in cases where the identification rate is less than the treatment rate that this assumption becomes invalid. In such cases, T_e can be said to be equal to the identification rate instead. This is true because mortality is not tied to being on the waiting list but from the onset of symptoms. In this way, the waiting list in our model can be thought of as the list of all people who need treatment, even if the NHS is unaware of them.

This model can be extended to predict mortality and time to clear a waiting list for non-constant T_e, but we do not expand on that here.

Supplement 2: Data

We calculate the increase in capacity due to conversions and operational changes as follows. Assume that we increase operations by 20% due to operational changes and convert 10% of all SAVR to TAVI. Also assume that for every three SAVR patients five TAVI patients can be processed. If we convert 10% of SAVR cases to TAVI (783 SAVR patients), we can treat an additional 522 patients from the waiting list. From the 20% increase, we get extra 1039 TAVI and 1566 SAVR operations per year. If we apply 10% conversion to this extra capacity, 156 SAVR operations can be converted into 260 TAVI operations. In total, the operational changes and conversion create an extra capacity of 3232 operations with which to service the waiting list each year: 1822 (1,039+522+261) TAVI and 1410 (1,566-156) SAVR operations.

N.B. We make no assumptions about who the extra TAVI procedures treat, for example, if in the above example, the additional 626 TAVI procedures we gain from conversion (522 from converting the normal capacity and 104 from converting the additional capacity) treated only SAVR patients, the conversion rate would actually be $\frac{626+783+156}{626+1566+7830} = 15.6\%$. Normally, we would expect that the application of this extra TAVI would be in the same proportion as the ratio of SAVR to TAVI, which would give a real-world conversion rate of 13.5%.

Supplement 3: App

The app can be accessed at https://github.com/Christian-P-Stickels/AS_Waitinglist_data

For peer review only

Supplement 4: Additional Results

Supplementary figure S1: Heat map of a three-to-four SAVR-to-TAVI conversion

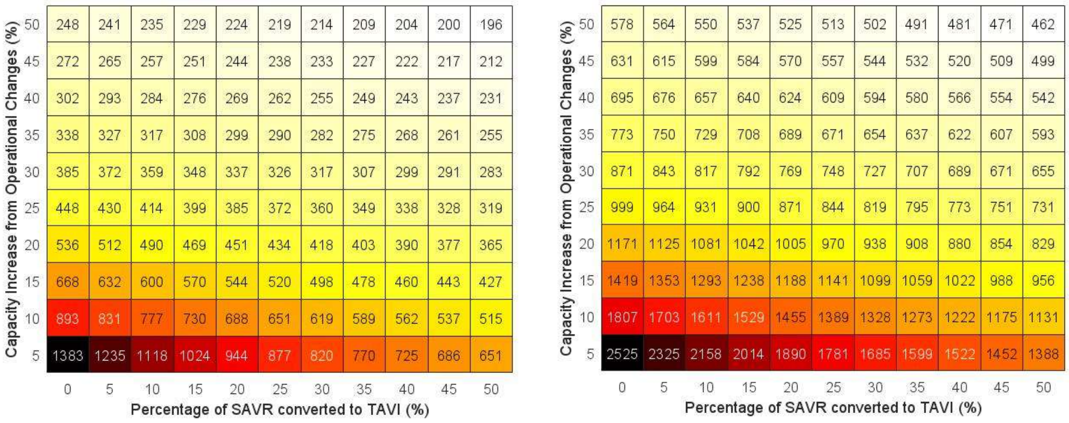

Supplementary Figure S1: Mean time to clear backlog (left) and the resulting deaths (right) as a function of daily percentage increase in capacity (y-axis) and percentage of SAVR converted to TAVI (x-axis), assuming that for every three SAVR operations, four TAVI procedures can be performed instead.

Supplementary figure S2: Heat map of a three-to-five SAVR-to-TAVI conversion

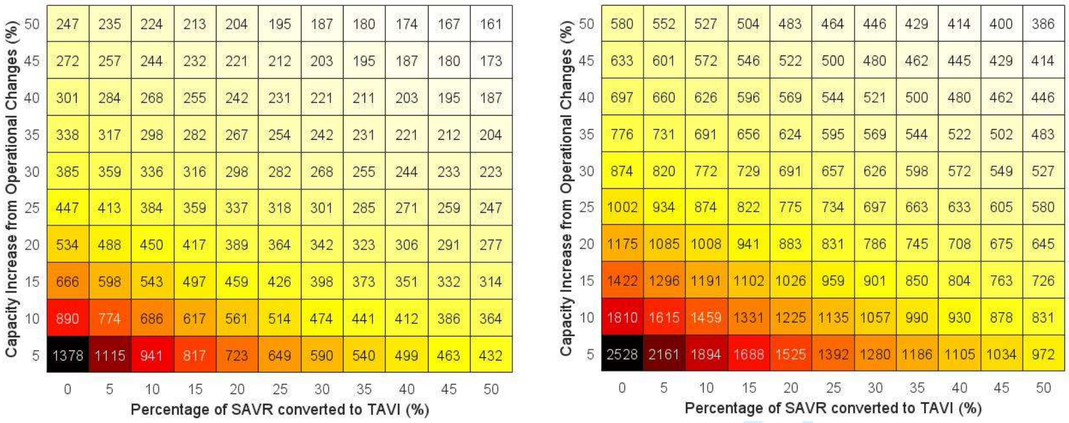

Supplementary Figure S2: Mean time to clear backlog (left) and the resulting deaths (right) as a function of daily percentage increase in capacity (y-axis) and percentage of SAVR converted to TAVI (x-axis), assuming that for every three SAVR operations, five TAVI procedures can be performed instead.

For peer review only - http://bmjopen.bmj.com/site/about/guidelines.xhtml

Supplementary figure S3: Heat map of a two-to-four SAVR-to-TAVI conversion

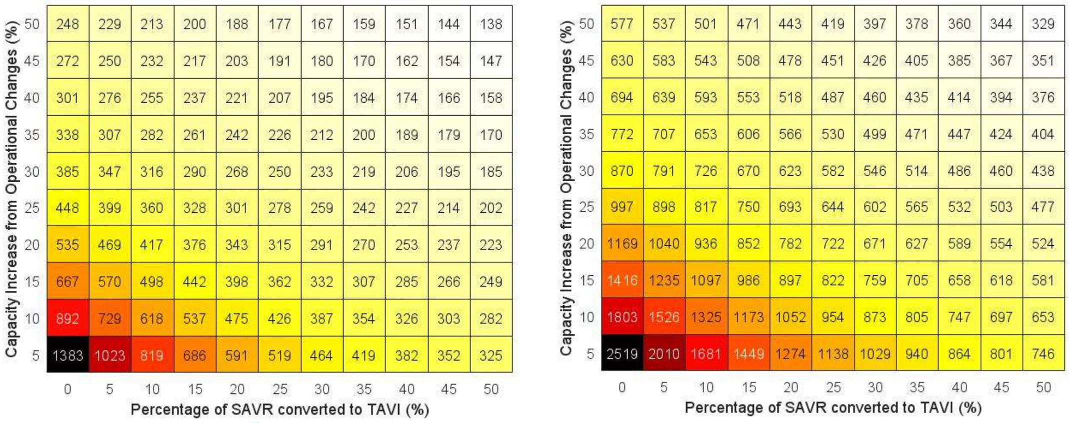

Supplementary Figure S3: Mean time to clear backlog (left) and the resulting deaths (right) as a function of daily percentage increase in capacity (y-axis) and percentage of SAVR converted to TAVI (x-axis), assuming that for every two SAVR operations, four TAVI procedures can be performed instead.

Supplementary figure S4: Error from mortality estimates

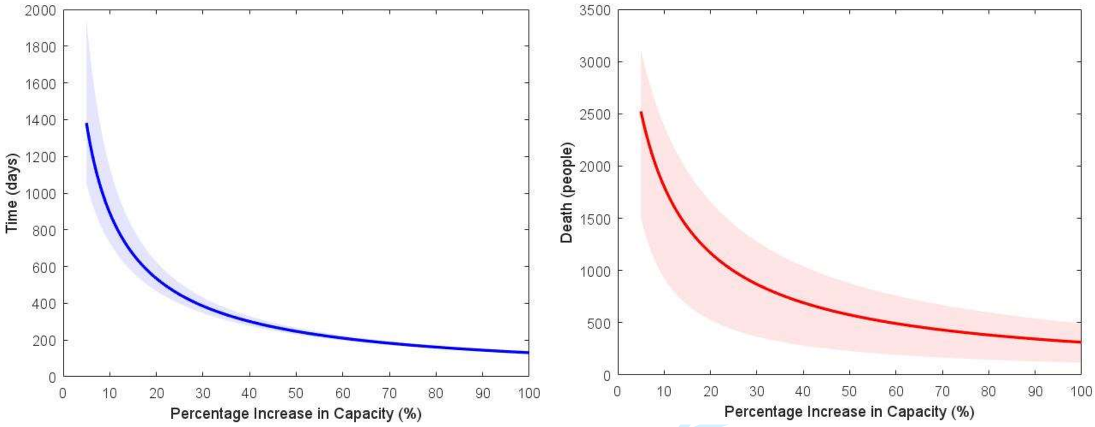

Supplementary figure S4: Time to clear backlog (left) and the resulting deaths (right) with associated 95% reference ranges as a function of daily percentage increase in capacity, with uncertainty from mortality only. The x-axis is truncated at 5% for visualisation and clarity.

We find that error in the one-year mortality causes higher uncertainty at lower capacity increases, but at higher capacity increases, this uncertainty decreases until it is almost zero with regards to clearance time. This is likely because at higher capacity increases, more of our waiting list clearance comes from treatment, as opposed to death, resulting in less error.

Supplementary figure S5: Error from wait list (W_0) estimates

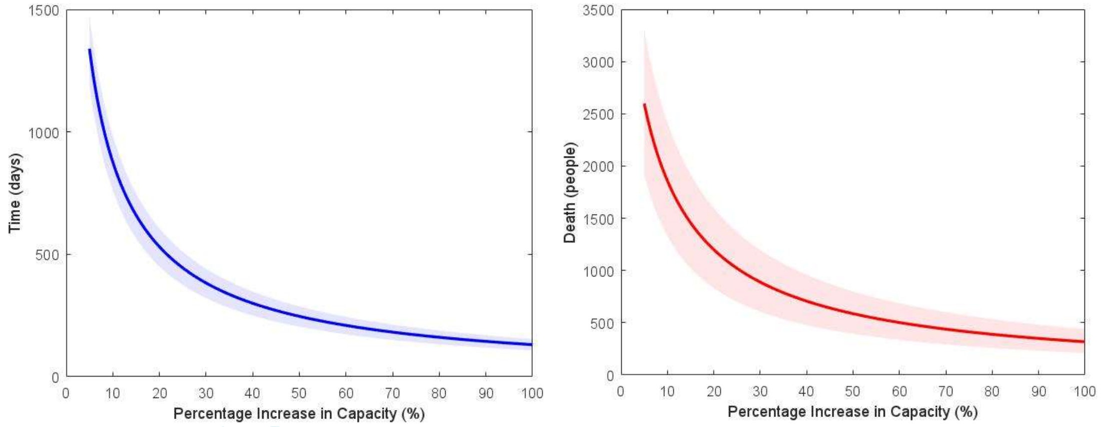

Supplementary figure S5: Time to clear backlog (left) and the resulting deaths (right) with associated 95% reference ranges as a function of daily percentage increase in capacity, with uncertainty from initial waiting list estimates only. The x-axis is truncated at 5% for visualisation and clarity.

We find that error in the estimate of the wait list length W_0 causes uncertainty that is fairly constant in the time it takes to clear the backlog and in resultant deaths. This is to be expected as we can show that the uncertainty scales with $\ln W_0$. There is a small decrease in uncertainty as we increase capacity, once again because an increase in capacity results in more control of the waiting list reduction.
